# Super-resolution multicolor fluorescence microscopy enabled by an apochromatic super-oscillatory lens with extended depth-of-focus

**DOI:** 10.1038/s41467-023-40725-9

**Published:** 2023-08-22

**Authors:** Wenli Li, Pei He, Dangyuan Lei, Yulong Fan, Yangtao Du, Bo Gao, Zhiqin Chu, Longqiu Li, Kaipeng Liu, Chengxu An, Weizheng Yuan, Yiting Yu

**Affiliations:** 1 Ningbo Institute of Northwestern Polytechnical University, College of Mechanical Engineering, Northwestern Polytechnical University, Xi’an, 710072 China; 2 Key Laboratory of Micro/Nano Systems for Aerospace (Ministry of Education), Northwestern Polytechnical University, Xi’an, 710072 China; 3 Shaanxi Province Key Laboratory of Micro and Nano Electro-Mechanical Systems, Northwestern Polytechnical University, Xi’an, 710072 China; 4 Department of Materials Science and Engineering, City University of Hong Kong, Hong Kong, 999077 China; 5 The Institute of AI and Robotics, Fudan University, Shanghai, 200433 China; 6 Key Laboratory of Spectral Imaging Technology of Chinese Academy of Sciences, Xi’an Institute of Optics and Precision Mechanics, Chinese Academy of Sciences, Xi’an, 710119 China; 7 Department of Electrical and Electronic Engineering, Joint Appointment with School of Biomedical Sciences, The University of Hong Kong, Hong Kong, 999077 China; 8 State Key Laboratory of Robotics and System, Harbin Institute of Technology, Harbin, Heilongjiang 150001 China

**Keywords:** Imaging and sensing, Biophotonics

## Abstract

Planar super-oscillatory lens (SOL), a far-field subwavelength-focusing diffractive device, holds great potential for achieving sub-diffraction-limit imaging at multiple wavelengths. However, conventional SOL devices suffer from a numerical-aperture-related intrinsic tradeoff among the depth of focus (DoF), chromatic dispersion and focusing spot size. Here, we apply a multi-objective genetic algorithm (GA) optimization approach to design an apochromatic binary-phase SOL having a prolonged DoF, customized working distance (WD), minimized main-lobe size, and suppressed side-lobe intensity. Experimental implementation demonstrates simultaneous focusing of blue, green and red light beams into an optical needle of ~0.5λ in diameter and DOF > 10λ at WD = 428 μm. By integrating this SOL device with a commercial fluorescence microscope, we perform, for the first time, three-dimensional super-resolution multicolor fluorescence imaging of the “unseen” fine structures of neurons. The present study provides not only a practical route to far-field multicolor super-resolution imaging but also a viable approach for constructing imaging systems avoiding complex sample positioning and unfavorable photobleaching.

## Abstract (web summary)

Conventional super-oscillatory devices suffer from numerical-aperture related issue including depth of focus, chromatic dispersion, and focusing, Here, the authors utilised multi-objective genetic algorithm to optimise the design and experimentally demonstrated lens with an extended depth of focus, ultra-large working distance and suppressed side-lobes.

## Introduction

Optical microscope, a basic tool for exploring and revealing the “secrets” of life science at the cellular and subcellular levels, often suffers from the resolving limit defined by the Abbe-Rayleigh diffraction limit. Ever-growing efforts have been dedicated to overcoming the diffraction limit, and relevant methods can be categorized into the near-field label-free and the far-field fluorescent-labeling approaches based on their respective working principles. In the near-field regime, the negative-index superlens^1,2^, field concentrators^3–5^, microsphere-assisted imaging^6,7^ and scanning near-field optical microscopy (SNOM)^8^ can resolve the fine details of an object located tens of nanometers from the lenses by invoking and collecting high spatial frequencies of evanescent waves. However, it has always been difficut to implement these approaches for practical applications in many scenarios, such as biological imaging, mainly due to the extremely small working distances (WDs). In the far-field regime, stimulated emission depletion (STED) microscopy^9,10^, photo-activated localization microscopy (PALM)^11,12^, and stochastic optical reconstruction microscopy (STORM)^13,14^ have been demonstrated to achieve super-resolution imaging at WDs of tens of micrometers. But these far-field super-resolution imaging methods are only applicable for labeled biological specimens, and often require the use of high-energy lasers, mercury or xenon lamps to selectively activate labeled fluorophores or single molecules, resulting in considerable photobleaching, fluorescence quenching and irreversible photodamage. In addition, some of these approaches involve complicated post-processing algorithms to obtain computed images that overcome the fundamental resolution limit of the optical lenses used. Notably, multicolor super-resolution imaging can enable spectral visualization of molecular interactions in biological samples^15–17^, but it still remains an intractable challenge for the above-mentioned far-field and near-field imaging techniques due to issues like chromatic aberration and limited depth-of-focus (DoF).

Recently, a non-invasive, label-free, far-field super-resolution imaging technique, called super-oscillatory lens (SOL) optical microscopy, has been proposed to address these issues^18^. Nevertheless, the SOL devices developed thus far still suffer from the following limitations: (1) short DoF resulting from highly compressed light fields by high numerical aperture (NA);^19–22^ (2) small field of view (FoV) limited by the strong side-lobes^23,24^; (3) chromatic aberration arising from the wavelength-dependent phase delay^19,25–27^; and (4) short WDs at the level of tens of micrometers^20,22,25–28^. Many efforts have been devoted to surmounting these inherent limitations and the corresponding results are summarized in Supplementary Table 1. Compared with conventional single-hotspot focusing patterns^19–27^, the central stop of phase plate^29,30^ and the vectorial polarization beams^31,32^ are applied to extend the DoF. Unfortunately, the former method sacrifices the focusing efficiency and degrades the imaging resolution in the transverse plane, and the latter can only yield hollow needles with DoF ≤ 10λ. Consequently, the best apochromatic two-dimensional (2D) planar SOL reported in the literature has a 1 ~ 2 μm long DoF and an extremely low focusing efficiency of <3%, making it unsuitable for realistic bio-imaging applications^19^. Although three-dimensional (3D) super-resolution imaging has recently been demonstrated with a DoF-extended SOL composed of non-subwavelength-sized chromium belts^28^, its WD, transmission efficiency and monochromaticity all need significant improvements for realistic cellular imaging applications.

To the best of our knowledge, the recently developed SOLs^22–28,33–37^ can only address one, or at most two of the four issues mentioned above. Grounded on our earlier studies^36,37^, here, we apply multi-objective genetic algorithm (GA) to address all the four issues simultaneously by devising an apochromatic binary-phase SOL with extended DoF, increased WD and multicolor super-resolution capability. This approach achieves successive focusing in the longitudinal direction, forming an optical needle with a DoF >10λ, which is orders of magnitude longer than the best results reported in the literature^19,36^. Multicolor super-resolution imaging can hence be realized by overlapping the optical needles of blue, green and red light beams, with a side-lobe suppression ratio of 10.13 dB, 10.66 dB and 9.32 dB respectively at a focal distance of 428 μm, surpassing all results published in the literature^20,21,29,30,33,37^. We finally showcase the application of the developed SOL on a fluorescence microscope for label-free 2D and 3D imaging, which reveals a far-field resolution limit of 0.53λ at 488 nm under continuous scanning and demonstrates its capacity of resolving sample’s in-plane information at different depths at one go without multiple out-of-plane scannings. More importantly, we successfully demonstrate for the first time, the multicolor fluorescence 3D imaging of labeled neuron tissues at different depths at one go. Such a versatile, lightweight and cost-effective high-NA SOL-based microscope will find promising applications in the multicolor super-resolution 3D imaging of label-free inorganic materials and labeled organic tissues, and thus significantly extend the scope of optical microscopy to the regimes that cannot otherwise be achieved by the commercially available confocal, multiphoton, STED, PALM and STORM microscopies. Importantly, the apochromatic SOL with extended DoF can also be applied in diverse fields, including super-resolution multicolor stereomicroscopy^38^, tunable spectral microscopy^39^, and targeted therapy beyond the diffraction limit^40^.

## Results

### Optimization of apochromatic SOL with extended DoF

**Fig. 1:**
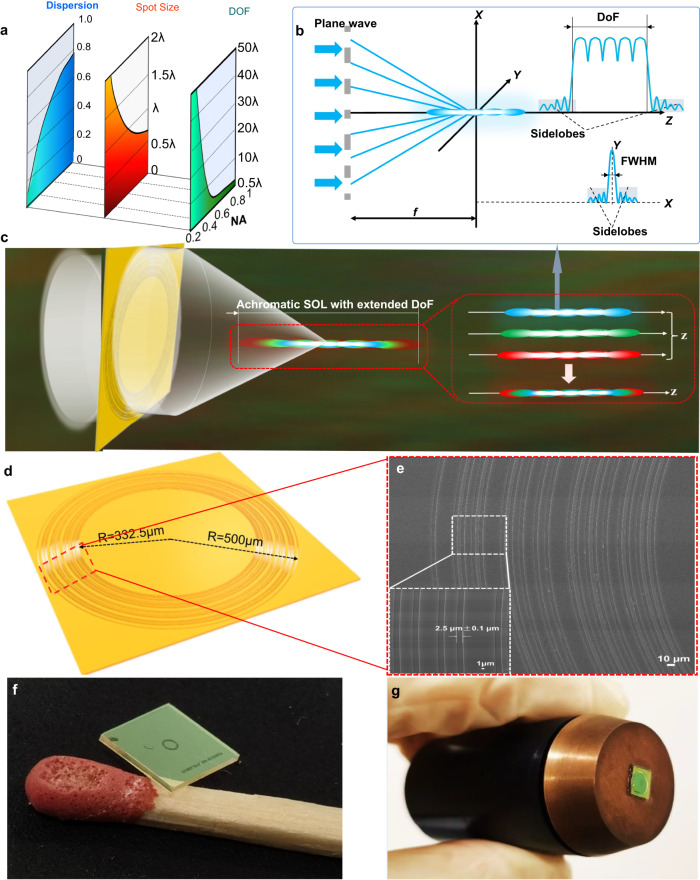
Design and realization of the proposed SOL. **a** NA-dependent tradeoff among the chromatic dispersion, focusing spot size and DoF of a diffractive lens such as a planar SOL. **b** Conceptual formation of a needle-like optical pattern by connecting adjacent multifoci under monochromatic illumination, with the minimized side-lobe intensity and main-lobe FWHM. **c** Schematic illustration of sub-diffraction-limit focusing by an apochromatic SOL with an extended DoF over the whole visible spectrum. The inset sketches the formation of an apochromatic sub-diffraction-limit optical needle by superposing the focus contours at the blue, green and red light wavelengths. **d** Schematic illustration of the proposed apochromatic SOL device drilled in a 215 nm-thick Si_x_N_y_ film. The innermost and outermost rings are 332.5 µm and 500 µm in radius, respectively. **e** Scanning electron microscopy (SEM) micrograph of the device with a zoom-in view shown in the inset. **f** Optical image of the fabricated SOL compared with a matchstick. **g** Optical image of a customized SOL-based objective.

The DoF of a conventional diffractive lens follows DoF = λ/[*n*(1-cos*α*_max_)], dictated by the optical analog of the uncertainty principle^41,42^. The largest convergence angle *α*_max_ between the outermost ray and the optical axis relates to the maximum spatial frequency of an imaging system with NA = n·sin*α*_max_. As a result, a larger NA gives rise to a compressed focusing spot (i.e. a smaller spot size) accompanied by a reduced DoF, as dipicted in the middle and right-hand panels of Fig. 1a. On the other hand, the increase of NA of a diffractive lens also incurs more significant chromatic abberation due to the increased dispersion associated with the reduced operational bandwidth (see the left-hand panel in Fig. 1)^43^. Therefore, there always exists an NA-dependent intrinsic tradeoff between the DoF, focusing spot size and chromatic abberation in conventional diffractive optical elements as well as in SOLs. To optimize this tradeoff for maximizing the NA-dependent DoF and simultaneously minimizing the chromatic dispersion and focusing spot size, here we apply a new multi-objective optimization strategy to design an apochromatic binary-phase SOL. The designed SOL owns an extended and highly uniform optical-needle-like focusing region with suppressed side-lobes. To realize such a multicolor SOL device, we first extend the DoF based on an axially joint multifoci approach and minimize the side-lobe intensity as well as the main-lobe full width at half maximum (FWHM) of the focusing needle for individual wavelengths, as examplified by the blue light shown in Fig. 1b. Then, the sub-diffraction needle-like optical patterns generated from three separate wavelengths (e.g. blue, green and red) are spatially overlapped with each other to give rise to an apochromatic SOL with an extended DoF and minimized FWHM, as depicted in Fig. 1c.

As presented by Eqs. M1 and M2, we consider a universal optimization model to construct an achromatic optical pattern with an extended DoF (See Methods, GA-based optical field design for design details). The GA process is triggered by initializing hundreds of individuals with random design parameters as the initial generation. Then, the individuals are screened by the objective functions G1, G2, and G3 in Eq. (1), serving as the fitness functions for optimizing the DoF, the focusing spot intensity uniformity and the main-lobe FWHM at each working wavelength, respectively, which are then weighted and summed up to formulate the multi-objective optimization to purposely address the above-mentioned intrinsic tradeoff faced by conventional SOLs. After that, screened individuals mutate and cross-over to generate the offspring. The GA loop will continue if the ending condition (i.e. the maximum iteration step set in the optimization process) is not fulfilled; Otherwise, the loop will end up with an optimal optical pattern and return to us with the corresponding phase mask. Detailed optimization iteration processes and corresponding results can be found in Supplementary Note 1 and Supplementary Figs. 1–2. Note that the optimization at three separate wavelengths and the extension of DoF (>10λ) achieved in this work do not represent the ultimate potential of our multi-objective GA-based optimization method. An even longer DoF of 23.4λ can be realized at 640 nm illumination as unveilled in Supplementary Fig. 3, with its optical characteristics shown in Supplementary Table 3 in Supplementary Information. Besides, it should be noted that the diameter of the present SOL is set as 1.0 mm in order to balance the SOL focusing performance and the avaliable computing resource, but in principle our design appoach is feasible to design apochromatic SOLs with even larger NAs (see the example of NA = 0.84 given in Fig. S4 in Supplementary Information).

The phase mask obtained by the GA optimization process is given in Supplementary Fig. 1. The designed SOL composed of concentric rings with varying radii perforated in a 215 nm-thick Si_x_N_y_ film conforming to the result of Supplementary Fig. 2, is verified by the SEM image, as shown in Fig. 1e. The millimeter-scale device is fabricated through the conventional optical lithography process (see Methods, Wafer level fabrication and Supplementary Fig. 5). Notably, the accuracy of fabrication can be controlled to 0.1 μm by the standard UV lithography steps, ensuring the required focusing performance of SOLs with minimal deviation from the prescribed design. The fabricated SOL compared with a common matchstick is displayed in Fig. 1f to demonstrate its millimeter-scale aperture. And the configuration of the fabricated SOL integrated onto a customized objective is revealed in Fig. 1g.

### Characterization of focusing performance

**Fig. 2:**
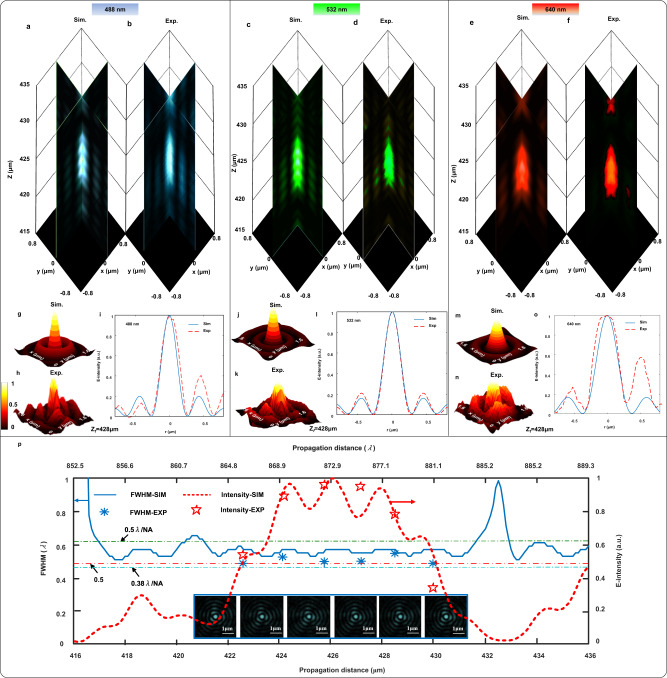
Apochromatic far-field subwavelength focusing. **a**, **c**, **e** Simulated and **b**, **d**, **f** experimental field patterns (in combined x-z and y-z longitudinal sections) at λ_B_ = 488 nm (**a**, **b**), λ_G_ = 532 nm (**c**, **d**), and λ_R_ = 640 nm (**e**, **f**) by an apochromatic SOL with extended DoF centered at z_f_ ≈ 428 μm. **g**, **j**, **m** Simulated and **h**, **k**, **n** measured intensity profiles in the transverse focal planes for the patterns in (**a**–**f**). Exp. experiment, Sim. simulation. **i**, **l**, **o** Comparison of the simulated and measured intensity profiles in the radial direction at z_f_ ≈ 428 μm. **p** Simulated (solid and dashed lines) and measured (asterisks and stars) FWHM and intensity of the optical needle’s main lobe along the propagation direction at λ_B_= 488 nm. The insets show the measured transverse intensity distribution profiles from *z* = 426 μm to *z* = 431 μm at a step size of 1 μm, each exhibiting a bright main lobe at the center and quite weak sidelobes around.

The theoretical calculation of a conventional diffractive lens with the same NA (0.76) shows the DoF of 1.4 μm at λ_B_ = 488 nm, 1.5 μm at λ_G_ = 532 nm, and 1.8 μm at λ_R_ = 640 nm, all of which are inferior to 3λ. To characterize the far-field focusing properties, fiber-coupled lasers at wavelengths of 488 nm, 532 nm and 640 nm with the linear polarization are used to illuminate the customized SOL from the substrate side. A Nikon inverted microscope is used to capture the light field (See Supplementary Note 3 and Supplementary Fig. 6 for the details of experimental validation of far-field focusing properties). The longitudinal cross-sectional distributions are formed from built-in data processing software ImageJ 1.47 v. The measured elongated hotspot within the range 422.9 ~ 430.1 μm at λ_B_ = 488 nm, overlaps with the hotspot range 425.1 ~ 432 μm at λ_G_ = 532 nm and 425.6 ~ 432.2 μm at λ_R_ = 640 nm, clearly revealing the significant DoF extension of our apochromatic SOL. The measured focal lengths of z_f_ ≈ 428 μm in Fig. 2b,d,f at the three wavelengths (488 nm, 532 nm and 640 nm) correlate well with the simulation results presented in Fig. 2a,c,e, validating the chromatic-dispersion-free property of this new SOL. Furthermore, the corresponding 3D views of the simulated and measured transverse intensity profile at z_*f*_ = 428 μm for the design wavelengths are respectively illustrated in Fig. 2g,j,m,h,k,n. The measured transverse modes verify the simulated results, implying that the intensity pattern is dominated by the central main hotspot without significant side-lobes. To quantitatively characterize the spot size of the created optical patterns, we plot both the simulated and measured intensity profiles at λ_B_, λ_G_ and λ_R_ in Fig. 2i,l,o, where good agreement is found between the simulated and measured results at λ_G_ = 532 nm, and slight differences exist for the remaining two wavelengths. The dissimilarity between the measured and simulated results is probably caused either by the imperfect integration of the SOL with the optical objective, or due to the lack of precision collimation between the objective and the incident optical excitation. More precise micro-electromechanical systems (MEMS) techniques may further improve the focusing quality at the aforementioned two wavelengths.

We tabulate the theoretical, simulated and measured results in Supplementary Table 2 to provide a direct proof of the advanced characteristics of this developed SOL. All the experimental results are the average of three measurements. Here, we define a figure of merit *V* = DoF_Sim_/DoF_Theory_ to quantify the DoF extension. An extension of at least 3.7 times for all three wavelengths is found, thus demonstrating the prominent DoF extension and the robustness of our design approach. The simulated and measured FWHM of the hotspots at three wavelengths: 245 nm (0.502 × λ_B_), 270 nm (0.507 × λ_G_) and 360 nm (0.562 × λ_R_) extracted from the simulated results and 256 nm (0.525 × λ_B_), 270 nm (0.507 × λ_G_) and 390 nm (0.609 × λ_R_) according to the measured results, obviously break the Abbe diffraction limit (0.66λ) with NA = 0.76. Other characteristic of our customized SOL, such as the side-lobe suppression ratio over 9.3 dB, is additionally presented, which surpasses the best reported apochromatic SOL with an extended depth-of-focus.

Moreover, we plot the curves of the measured FWHMs at different axial distances to investigate the uniformity of the designed optical needle-like focusing pattern. As demonstrated in Fig. 2p, the FWHMs break through the diffraction limit at five typical points within the focusing region at discrete axial distances which are extracted and marked as blue asterisks in bold at 488 nm (see Supplementary Note 4 and Supplementary Fig. 7 for properties at all three wavelengths).

The optical super-oscillation is the result of the delicate interference of far-field propagating waves, which can be accurately engineered to achieve sub-diffraction-limit focus preserving the fine physical details of objects^44,45^. The propagation region of interest (RoI) is colored within the range from 0.38λ/NA to 0.5λ/NA to highlight the DoF extension of super-resolution focusing at different incident wavelengths. Although there are some intensity fluctuations within the optical needle, the optical energy is confined within the sub-diffraction-limit region for all the three incident wavelengths. Taking λ_G_ as an example, five typical points at discrete propagation distances from z = 425 μm to z = 432 μm at a step of 1 μm are extracted, and the corresponding FWHMs measured are evaluated as 0.4511λ_G_, 0.5075λ_G_, 0.5075λ_G_, 0.5075λ_G_ and 0.5075λ_G_, respectively, all breaking the Abbe diffraction limit. Henceforth, the focusing spot sizes and contours are quasi-uniform within the customized needle-like focal region from z = 425 μm to z = 432 μm. This could greatly benefit the practical bio-imaging applications.

### Sub-diffraction multicolor 2D imaging

**Fig. 3:**
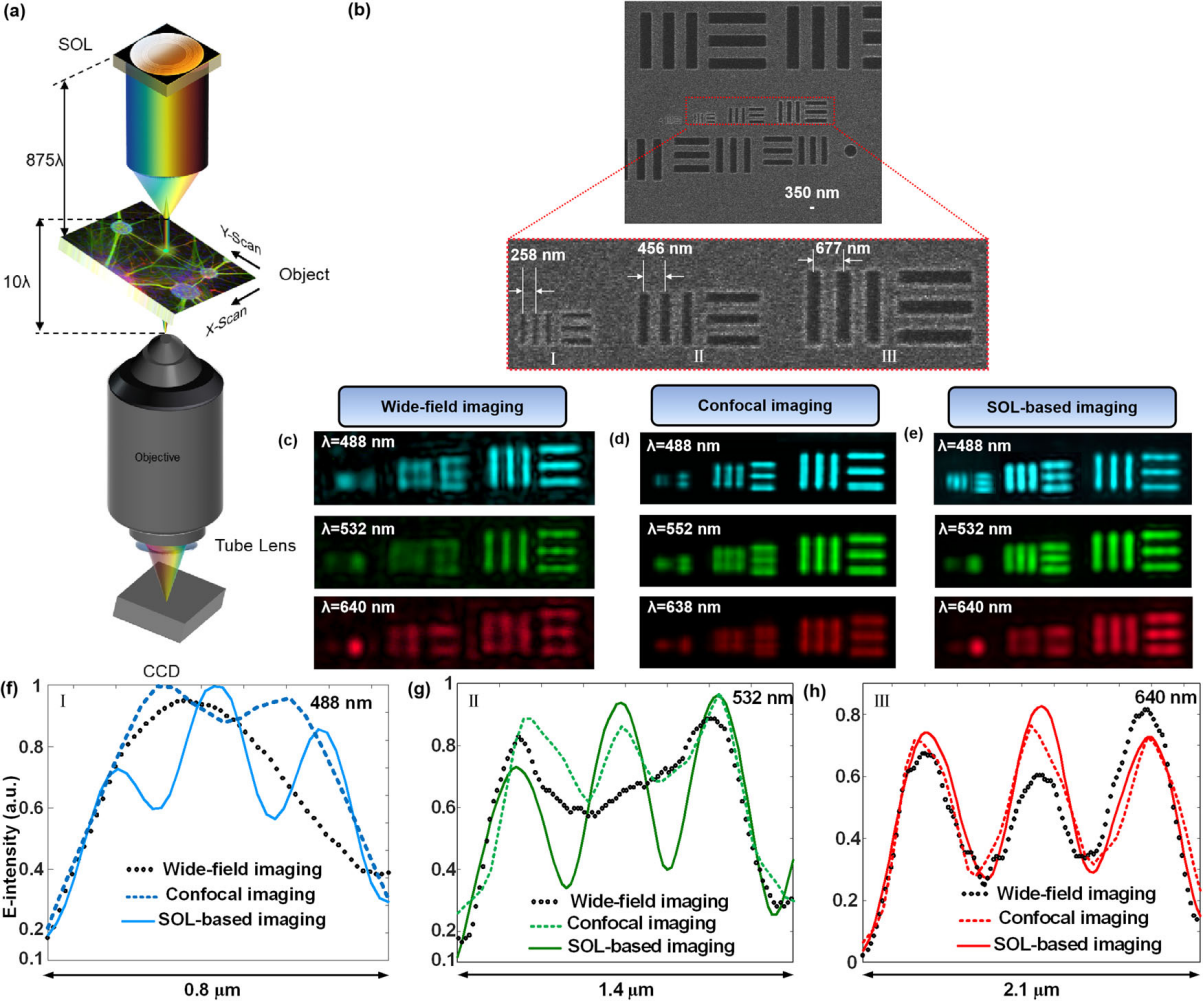
Sub-diffraction multicolor 2D imaging. **a** Sketch of an SOL-based scanning optical microscope. When the SOL is illuminated by a collimated laser beam, a subwavelength-focused, 10λ-long optical needle is formed at 428 μm away from the SOL output plane. A sample is placed within the needle length and scanned in the x-y plane. The transmitted light is collected by an objective and sent to a CCD camera for recording its image. **b** SEM micrographs of the resolution testing chart. The practical fabricated CTC distances between the slits in three structures (I-III) are 258 nm, 456 nm, and 677 nm, respectively. **c** Wide-field, **d** LSCM and **e** SOL-based imaging results for the testing chart in **b**. **f**–**h** Line-scanning intensity profiles of the experimental images by addressing the CTC distances from 150 to 350 nm at an interval of 100 nm with three different microscopes under three illumination wavelengths. The resolvable CTC distances are 677 nm for the traditional wide-field microscope, 456 nm for LSCM and 258 nm (0.53λ) for our SOL-based microscope at the incident wavelength of λ_B_ = 488 nm.

Based on the optimized apochromatic SOL with an extended DoF, we establish a customized high-resolution multicolor microscopy system for the scanning mode of high-quality imaging applications with a large FoV. To test its resolving capability, a homemade nanometer-scale resolution target is utilized. The imaging target is fabricated on a 100 nm-thick chromium layer by focused ion beam (FIB) milling. The working principle of the customized imaging system is sketched in Fig. 3a, in which the apochromatic SOL is used to provide a sub-diffraction-limited needle-like optical contour along the z axis. A piezoelectric transducer provides precise mechanical movements to ensure the relative displacement between the sample and the optical needle (see Supplementary Note 5 and Supplementary Figs. 8-9 for the imaging setup and the process). The measured center to center (CTC) distances of the resolution testing chart are 258 nm, 456 nm and 677 nm, respectively, as shown in Fig. 3b. Noting that the CTC distance of the closest-spaced slits is beyond the diffraction limit for the three visible light wavelengths. This testing chart is first examined under a conventional inverted microscope (objective NA = 0.9) without the SOL in the transmission mode. The transmitted light signals from the testing chart under respective illumination of three incident wavelengths are mapped in vague appearances, where many slits cannot be resolved (Fig. 3c). Slightly improved results can be obtained by a laser scanning confocal microscope (LSCM), as shown in Fig. 3d, but the 488 nm illumination fails to resolve the six closest-spaced slits either. In contrast, our apochromatic SOL-based microscope is able to reveal the finest separations of these slits, and even the 258 nm CTC distance can be resolved under 488 nm illumination (Fig. 3f). To alleviate the effect of sidelobes on the imaging results and simplify the whole imaging setup, the sidelobe-main lobe intensity ratio at the focal plane is purposely set to be below 0.3 in the GA optimization process. During the imaging process, all the image information within the FoV of the objective is collected by the built-in charge-coupled device (CCD) camera. To reconstruct the final complete images, the template matching algorithm is applied and then the serial images are merged together. The cucoloris of the scanning imaging process for the resolution test target at the illumination of λ = 640 nm is given in Supplementary Movie 1. The ultimate reconstructed images of the slits from 150 nm to 350 nm at the three illumination wavelengths (Fig. 3e) also show that our SOL-based microscope could achieve a high-quality image and reveal the objects with smaller distortion at a low cost. The quantitative comparison between the line-scanning intensity profiles shown in Fig. 3f confirms that our SOL-based microscope exhibits a resolution of 258 nm (i.e., 0.53λ at λ = 488 nm). The peak-valley intensity contrast in the profiles recorded by our extended-DoF SOL microscope ranges from 40% to 50% under the three illumination wavelengths (Fig. 3e-h). Such contrast is substantially higher than that of 20% as defined by Rayleigh resolution criterion for distinguishing two neighboring points in one image^46^. Based on the point scanning imaging mode, narrower slits etched in the metallic layer will be resolved via the customized SOL-based optical microscope.

### Axial-scanning-free 3D imaging

**Fig. 4:**
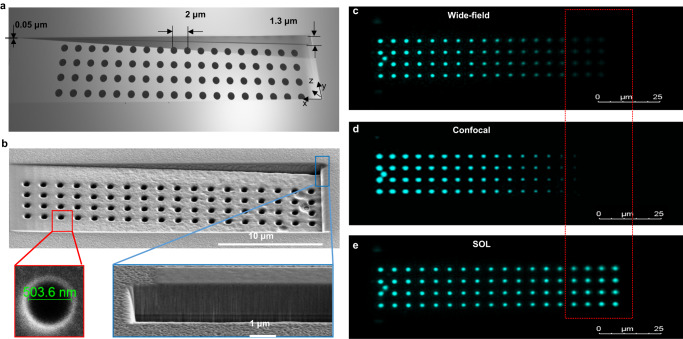
Axial-scanning-free imaging of a 3D object. **a** Sketch of a 3D metallic fishnet wedge composed of an array of circular holes with a period of 2 μm. The depth of the wedge varies from 0.05 μm at the left edge to 1.3 um at the right edge, and the diameter of the holes is about 500 nm. **b** Top-view (x-y plane) SEM micrograph of a fabricated fishnet wedge. The two insets at the bottom are the zoom-in images of a single hole (left panel) and the top-right cornner of wedge. **c**–**e** Images of the wedge in **b** collected by the wide-field microsocpe **c**, LSCM **d**, and our DOF-extended SOL-based microscope all working in the transmission mode (T-mode).

One unique advantage of the DoF-extended SOL-based microscopy is the capability of imaging the details of a 3D object at one go, without multiple out-of-plane axial scannings. Figure 4a,b shows the schematic and SEM micrograph of a 3D metallic wedge composed of an array of circular holes drilled in an opaque metal plate, similar to the sample studied in Ref. ^28^. This wedge has an oblique angle *α* ≈ 43.3 mrad and results in a height difference of ~1.3 μm between its left and right sides. As shown in Fig. 4c,d, both the conventional T-mode wide-field microscope and the LSCM can only image part of the holes at a certain z-cut plane because their short DoFs cannot cover all the holes at different depths. Therefore, multiple out-of-plane axial scannings must be performed for the T-mode wide-field microscope and the LSCM in order to reconstruct all the in-plane (x-y plane) details of this 3D object. In contrast, the imaging result by our DoF-extended SOL-based microscope presented in Fig. 4e shows a clear mapping of this array by precisely addressing the positions of each hole. When the SOL-generated optical needle illuminates the sample, a series of images can be captured by a detector and then stitched together to form a whole image of the sample. The superiority of our SOL-based microscope is highlighted by the red dashed box in Fig. 4c-e (See Supplementary Note 6 and Supplementary Fig. 9 for the details of experimental validation of far-field focusing properties).

### Dual-color 3D cellular imaging

**Fig. 5:**
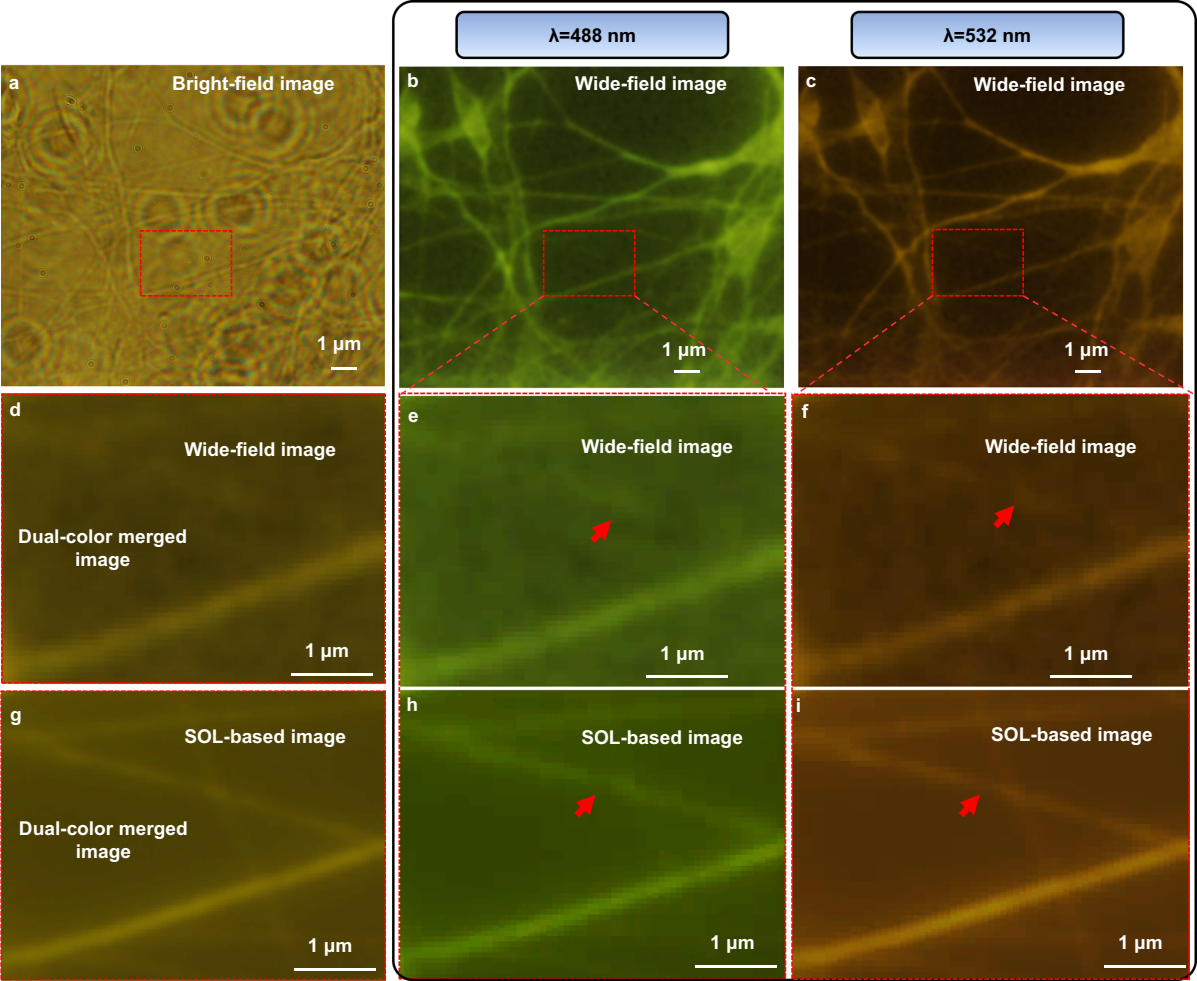
3D dual-color cellular imaging. **a** Bright-field and **b**, **c** Wide-field fluorescence microscopy images of a thick neuron tissue. **e**, **f** Zoom-in views of the enclosed areas in **b**, **c**. **h**, **i** SOL-based microscopy images of the enclosed area in **b**, **c**. **d**, **g** Merged dual-color fluorescence images from wide-field imaging and SOL-based microscope. The red arrows in **e**, **f**, **h**, **i** denote the fine structures of nuerons.

Imaging biological samples with a certain thickness is usually limited by an inevitable obliquity between the sample and lens plane. This often leads to out-of-focus blurred images and therefore limits the FoV of imaging systems with a short DoF. When observing the internal fine structures of neurons where internal synapses always lie at different depths, several key capabilities such as a high spatial resolution, an extended DoF and apochromatic property are required. Mapping the activity of neurons at a high resolution can provide a great insight into how neuronal ensembles collectively drive brain function for researchers. To promote the low-cost multicolor super-resolution stereo fluorescent imaging application of the SOLs, the apochromatic DoF-extended SOL-based microscope is employed to observe the human neurons cultured and fixed on the 170 μm-thick glass slide. The favorable improvement brought about by the cover glass and water on the focusing properties, e.g. an even extended DoF and smaller focusing spot, is numerically revealed in Supplementary Table 4. The cell culture and fluorescent labeling information are available in the Supplementary Materials section. The excitation and emission wavelengths of the two fluorescent dyes are 488 nm & 520 nm and 546 nm & 580 nm, respectively (see Supplementary Note 7 and Supplementary Fig. 10 for the details of dual-color 3D cellular imaging). Figure 5a displays the cellular image captured by the bright-field microscope, and the RoI region is highlighted in the red dotted box. The wide-field fluorescent images are showcased in Fig. 5b,c and the zoom-in figures of the wide-field fluorescent images are demonstrated in Fig. 5e,f. Note that the short DoF of the commercial imaging objective is not able to cover the neuronal synapse at different depths. Therefore, the whole fine details within RoI can only be obtained through several out-of-plane movements of the objectives. This multiple positioning will incur inevitable photobleaching and make the imaging process more complicated. Compared with the wide-field fluorescent results, the blurred morphologies can be distinctly mapped by the SOL-based microscope for the two incident wavelengths, as shown in Fig. 5h,i. To characterize the multicolor fusion results, the two images obtained at two incident wavelengths are merged, as shown in Fig. 5d,g. The 3D information of the neurons at different depths can be observed more clearly at one go by in-plane scanning of the SOL. The detailed splicing process is introduced in Methods, Splicing of fluorescent-labeled cell images and the cucoloris of the scanning imaging process for the neuron cell at the illumination of λ = 488 nm can be seen in Supplementary Movie 2. The final cellular imaging results demonstrate that our customized apochromatic SOL-based optical microscopy (SOM) can realize the far-field multicolor sub-diffraction-limit cellular imaging with a large DoF.

## Discussion

For the real-life high-resolution multicolor fluorescent imaging applications, the sub-diffraction-limit dispersion-corrected hotspot with an extended DoF is needed to realize the clear and informative imaging results. SOL-based optical microscope is a highly customized, non-invasive and universal imaging technique without complex mathematical post-processing. To develop the high-NA apochromatic SOL with an extended DOF from the perspective of structural innovation and algorithm improvement, the axially jointed multifoci method is proposed in this work. Based on the proposed method, the SOL (NA = 0.76) with an extended DoF and the same WDs for three discrete incident wavelengths is optimally designed. The later experimental measurements demonstrate a good correlation with the simulated results for the proposed SOL. As expected, both the designed and measured DoFs exceed the theoretically results defined by the conventional diffractive lenses with the same NA. Furthermore, the ultra-long and identical WD surpasses the best reported result in literature. Finally, all the FWHMs of hotspots at three wavelengths successfully break the Abbe diffraction limit. As a result, all these features of the newly developed SOL allow its integration into a customized microscope to perform super-resolution multicolor imaging applications. It should also be pointed out that our optimized SOL can only work at three discrete wavelengths without bandpass filters or any mechanical out-of-plane movement^47^. SOL devices made of dispersionless structures can be considered to make it work under white light illumination in the future^48^.

Then we exploit our SOL-based multicolor fluorescent microscope in the practical label-free and labeled imaging capability. Firstly, the resolving power is tested by the customized nanoscale resolving target. At the incident wavelength of 488 nm, the 258 nm nanoslits can be easily distinguished, in contrast to the blurred images formed by the wide-field imaging microscope. To demonstrate the practical imaging ability with the extended DoF, the apochromatic SOL is employed to observe human neurons labeled by the two dyes simultaneously. Three discrete neurons synapses at different observing planes within the RoI area are selected as the main structures. Compared with the direct wide-field fluorescent imaging results generated from the high-NA (NA = 0.9) objective, the contours can be better distinctly mapped, which shows the desired extended DoF of the apochromatic SOL as proposed. Additionally, based on the flexible and controllable design method, the energy ratio for the focusing hotspot of the SOL can be further adjusted to satisfy various samples without damaging the biological activity, which greatly helps to build up the customized optical imaging system. Compared with the expensive confocal and multiphoton microscopes, the apochromatic SOL-based microscope possesses a higher resolution with an extensively DoF which is at a low cost and with greater flexibility. Admittedly, the bare imaging results provided by the apochromatic SOL-based microscopy system shows an inevitable background noise. To solve this problem, the noise reduction and template matching algorithms are employed to reconstruct the imaging results. It is believed that the results can be further improved by updating the used CCD camera with a higher yield. In addition, the DoF for the apochromatic SOL can be extended to tens of micrometers by the further algorithm optimization.

The high-NA SOL with the apochromatic extended DoF will promote the practical applications for the planar SOLs in many fields, such as non-invasive 3D biomedical imaging, laser tweezing, multicolor optical coherence tomography imaging, spectral microscopy imaging, lab-on-chip devices and micro/nanofabrication. The unique advantage of the customized light-field patterns for the SOLs in the far field will make biological imaging much more flexible and efficient.

## Methods

### GA-based optical field design

In our theoretical design, the genetic algorithm (GA), a powerful computational tool to solve multi-objective and multi-constraint optimization problems and generate Pareto-optimal solutions^22,49–51^, is employed to optimize the DoF of SOL while minimizing the side-lobe intensities and the main-lobe FWHM under monochromatic illumination. Since a subwavelength-sized optical field pattern can be constructed by the interference caused by an SOL consisting of multiple concentric microrings, in the GA-based SOL design. The outer radius, widths of the concentric rings, and locations of discrete foci along the optical axis are fixed for specific incident wavelengths while the central positions of the rings are set as variables. To produce a large and uniform DoF, the number of desired discrete axial focal spots *M* are initially preset, and these focal hotspots are then gradually prolonged to overlap with each other; To reduce the side-lobe intensities and shrink the main-lobe FWHM, we restrain the field pattern’s intensities along two orthogonal directions perpendicular to the optical axis. The intensity ratio between the side-lobes and the central focusing spot is set to be below 0.3 in order to produce a sharp contrast. This constraint ensures a high signal-to-noise ratio, favorable for fluorescent imaging by selectively illuminating biological tissues exclusively with the central focusing hotspot. With the aforementioned methodology, an optimization model is developed based on Eq. (1), which is subject to the conditions given in Eq. (2). The DoF, side-lobe intensity and main-lobe FWHM at different foci along the optical axis are simultaneously optimized by the three objective functions as follows:

1 $$\begin{array}{c}{G}_{1}=\,\max \left[I(0,{f}_{1-};{t}_{i};{\lambda }_{q}),I({f}_{M+},z;{t}_{i};{\lambda }_{q})\right];\\ {G}_{2}=\,\max \left|I({f}_{m-},{f}_{m+};{t}_{i};{\lambda }_{q})-I({f}_{1-},{f}_{1+};{t}_{i};{\lambda }_{q})\right|;\\ {G}_{3}=I(\frac{{{{{{\rm{FWHM}}}}}}}{2},{f}_{m-};{t}_{i};{\lambda }_{q});\\ \min {G}_{1},{G}_{2},{G}_{3}\end{array}$$

s.t.

2 $$\begin{array}{c}{f}_{n+}={f}_{(n+1)-};\\ \bigg|\begin{array}{cc}I(0,\,{z}_{0};{t}_{i};{\lambda }_{q}) & 1.15\\ 1 & 1\end{array}\bigg|\le 0\le \bigg|\begin{array}{cc}I(0,\,{z}_{0};{t}_{i};{\lambda }_{q}) & 0.95\\ 1 & 1\end{array}\bigg|;\\ \bigg|\begin{array}{cc}I(0,\, {z}_{x};{t}_{i};{\lambda }_{q}) & 0.3\\ 1 & 1\end{array}\bigg|\le 0;\\ \bigg|\begin{array}{cc}I(r,\,{f}_{m};{t}_{i};{\lambda }_{q}) & 0.3\\ 1 & 1\end{array}\bigg|\le 0,\bigg|\begin{array}{cc}r & k\frac{{{{{{\rm{FWHM}}}}}}}{2}\\ 1 & 1\end{array}\bigg|\le 0\le \bigg|\begin{array}{cc}r & \frac{{{{{{\rm{FWHM}}}}}}}{2}\\ 1 & 1\end{array}\bigg|;\\ {z}_{0}\in \left({f}_{1}-\frac{{\varDelta }_{f}}{2},\,{f}_{M}+\frac{{\varDelta }_{f}}{2}\right);\\ {z}_{x}\in \left(0,\,{f}_{1}-\frac{{\varDelta }_{f}}{2}\right)\cup \left({f}_{n}+\frac{{\varDelta }_{f}}{2},\,{f}_{n+1}-\frac{{\varDelta }_{f}}{2}\right)\cup \left({f}_{M}+\frac{{\varDelta }_{f}}{2},\, z \right);\\ q=1,\, 2,\cdots,Q;m=1,\, 2,\cdots,\, M;n=1,\, 2,\cdots,\, M-1;\\ {t}_{i}\in \{0,\, 1\},\, i=1,\, 2,\cdots,\, N;\end{array}$$

where *I* stands for the normalized field intensity of the needle-like focusing region; *λ*_*q*_ is the incident wavelength; *f*_*n*+_ = *f*_*n*_ + Δ_*f*_/2, *f*_(*n*+1)-_ = *f*_*n*+1_ − Δ_*f*_/2; *f*_*m*-_ = *f*_*m*_ − Δ_*f*_/2 and *f*_*m*+_ = *f*_*m*_ + Δ_*f*_/2, with *f*_*m*_ being the focal distance of the *m*-th hotspot and Δ_*f*_, the DoF of each hotspot; *Q* is the total number of incident wavelengths of interest; *t*_i_ is the transmittance value of the *i*-th annular ring and *N* represents the total number of concentric rings in the mask. The first objective function *min*(*G*_1_) seeks the energy surrounding each main axial focal spot to be as low as possible, with the aim to ensure the maximum intensity at the main focal spot; the second objective function *min*(*G*_2_) minimizes the difference in the intensities of all hotspots; the third objective function *min*(*G*_3_) minimize the FWHM of the main lobe, ensuring high resolution. Additionally, in order to generate a highly uniform main lobe, the normalized intensity over the focusing region is set to be within 0.95 and 1.05, and the axial intensity of other secondary points is set to be below 0.3 to ensure a sharp intensity contrast between the side and main lobes. To construct an unconstrained optimization process, the intensity modulation of the needle-like focusing pattern formed by merging adjacent axial multifoci is added as a penalty function term. To accelerate the numerical calculation, a fast Hankel transform algorithm is employed to ensure high accuracy, fast optimization, and less data storage^52^. Five main axial hotspots (*M* = 5) are chosen in this work, giving rise to the phase distribution profile of the optimized SOL shown in Supplementary Fig. 2. Wafer-scale nanofabrication. Parallel fabrication of optical elements, the main trend fabrication method compared to the complex and time-consuming processes such as electron beam lithography (EBL) and focused ion beam milling (FIB)^19,24,26–28,35^, has been employed in our work owing to its micrometric feature size. For the sake of practical application of the planar SOLs integrated with the on-chip optical imaging system, a functionalized SOL with 1-mm diameter is efficiently fabricated in a mass production way. The 2.5-μm feature size of the annuli of our optimized SOL makes it possible to fabricate the millimeter-scale device through the conventional optical lithography process^36,37^. The SixNy film is deposited by PECVD, followed by optical lithography used for graphic processing and RIE for etching. The fabrication process and the optical refractive index of SixNy film are presented in Supplementary Note 2 and Fig. S5.

To design a realistic dielectric apochromatic lens, the phase modulation difference induced by material dispersion ( > π for short wavelengths, <π for longer wavelengths) should be taken into consideration in the optimization process. For example, with an etching depth of 213 nm, a step in the Si_x_N_y_ layer creates a phase retardation of 1.1π at λ_B_ = 488 nm and 0.8π at λ_R_ = 640 nm. The phase deceleration related with material dispersion is included as additional phase in our GA optimization process.

### Splicing of fluorescent-labeled cell images

Different from the images captured from the resolution test target in high contrast, the imaging process of the cell fluorescent images is challenging especially for the feature matching. In this part, we make an introduction of the feature matching and image fusion process of the sequence images. Since affine transformation will occur between sequence images, we refer to the method of panoramic image Mosaic^53^ to estimate affine matrix. Compared with the extraction effects of ORB^54^, FAST^55^, SURF^56^ and other key points, SIFT key points^57^ show excellent translation and rotation invariant properties. The detailed image processing results for the four methods can be found in Supplementary Fig. 11. Henceforth, SIFT key points algorithm are chosen to complete the image matching so that the cell texture features could be clearly reconstructed. The KD-Tree^58^ is applied to select the most appropriate matching objects, in $O(n\,\log n)$ time, where the SIFT points and the affine matrix *H* is estimated^53^.

3 $${H}_{ij}={K}_{i}{R}_{i}{R}_{j}^{T}{K}_{j}^{-1}$$

where the camera parameter matrices *K* and *R* are defined as follows

4 $${K}_{i}=\left[\begin{array}{ccc}{f}_{i} & 0 & 0\\ 0 & {f}_{i} & 0\\ 0 & 0 & 1\end{array}\right]$$

5 $${R}_{i}={e}^{[{\theta }_{i}]_{X}},\,{[{\theta }_{i}]}_{X}=\left[\begin{array}{ccc}0 & -{\theta }_{i3} & -{\theta }_{i2}\\ -{\theta }_{i3} & 0 & -{\theta }_{i1}\\ -{\theta }_{i2} & {\theta }_{i1} & 0\end{array}\right]$$

To improve the accuracy of the affine transformation matrix *H* estimation, RANSAC^59^ algorithm is used to estimate the interior points in the matched SIFT point pairs. Then Levenberg-Marquardt algorithm is utilized to calculate the least square fitting for multiple point pairs (>4) to get the transformation matrix *H*. Referring to the methods mentioned in Ref. ^53^ and Ref. ^60^, the spatial consistency of multiple images is explored and the accumulated errors are reduced. In particular, the Bundle Adjustment method is adopted to process all images simultaneously, and the updated equation (Eq. (4)) is iteratively solved to obtain a more accurate transformation matrix.

6 $$\varPhi={({J}^{T}J+\lambda {C}_{p}^{-1})}^{-1}{J}^{T}r$$

where *Ф* are all the parameters, *r* the residuals and *J* = ∂r/∂*Ф*, the (diagonal) covariance matrix *C*_*p*_ is

7 $${C}_{p}=\left[\begin{array}{cccccc}{\sigma }_{\theta }^{2} & 0 & 0 & 0 & 0 & \cdots \\ 0 & {\sigma }_{\theta }^{2} & 0 & 0 & 0 & \cdots \\ 0 & 0 & {\sigma }_{\theta }^{2} & 0 & 0 & \cdots \\ 0 & 0 & 0 & {\sigma }_{f}^{2} & 0 & \cdots \\ 0 & 0 & 0 & 0 & {\sigma }_{0}^{2} & \cdots \\ \vdots & \vdots & \vdots & \vdots & \ddots & \cdots \end{array}\right]$$

Considering the environmental vibration and laser instability which may cause the difference in illumination conditions for each picture, a sharp gradient changes is noticeable in some areas during the fusion. Gain compensations are therefore introduced, resulting in the error function (Eq. (6)) to be constructed as the sum of the gain times and the normalized intensity errors of all overlapped pixels.

8 $$e=\frac{1}{2}\mathop{\sum }\limits_{i=1}^{n}\mathop{\sum }\limits_{j=1}^{n}\mathop{\sum}\limits_{{{u}_{i}\in \Re (i,j)\atop {{\tilde{u}}_{i}={H}_{ij}{\tilde{u}}_{j}}}}({g}_{i}{I}_{i}({u}_{i})-{g}_{j}{I}_{j}({u}_{j}))^{2}$$

where ${g}_{i}$, ${g}_{j}$ are the gains, and ${\mathcal R} (i,j)$ is the overlapped region between images *i* and *j*. Here a variable coefficient average method is applied to replace the same mean for the global images. To obtain the final fusion results, it is the necessary to track the central spot and enhance the clarity of the scanned texture of the central spot. The sub-regional average approximation is adopted here, *I*_*i*_(u_*i*_) can be approximated by ${\bar{I}}_{{ij}}$:

9 $$\overline{{I}_{ij}}=\frac{\mathop{\sum}\limits_{{u}_{i}\in \Re (i,j)}{I}_{i}({u}_{i})}{\mathop{\sum}\limits_{{u}_{i}\in \Re (i,j)}\alpha },\, \alpha=\left\{\begin{array}{c}0.8,\,I(\Re (i,\, j)) \, > \, 0.85\,\max \{I(\Re (i,\, j))\}\\ 1,\, I(\Re (i,\, j))\le 0.85\,\max \{I(\Re (i,\, j))\}\end{array}\right.$$

### Preparation of cell samples

The DYR0100 cells (induced pluripotent stem cells-iPSC) were provided by the research group of Prof. Cao Yi from Xiangtan University. Zhejiang Hopstem Bioengineering company limited provided the services of cell differentiation and labeling according to the method mentioned in the published article [DOI: 10.1126/scitranslmed.aad0623], which was used for bioimaging in this study.

### Statistics and reproducibility

No statistical method was used to deal with the experimental data. The experiments were repeated at least three times independently with similar results.

### Reporting summary

Further information on research design is available in the Nature Portfolio Reporting Summary linked to this article.

### Supplementary information

Supplementary Information

Description of Additional Supplementary Files

Supplementary Movie 1

Supplementary Movie 2

Reporting Summary

## Data Availability

The authors declare that all the data supporting the findings of this study are available within this paper and its Supplementary Information file, or available from the corresponding author Yiting Yu upon request. Considering the large size of imaging series of the super-resolution image files, the raw datasets are available from the corresponding author Yiting Yu on request. Requests will be answered within 3 weeks to arrange file transfer.

## Code availability

The multi-objective GA optimization is available on request from yyt@nwpu.edu.cn as the terms of the license established prior to this study with the Northwestern Polytechnical University and the Key Laboratory of Micro/Nano Systems for Aerospace (Ministry of Education) do not allow uploading the code in a public repository.

## References

[1] Pendry JB (2000). Negative refraction makes a perfect lens. Phys. Rev. Lett..

[2] Zhang X, Liu Z (2008). Superlenses to overcome the diffraction limit. Nat. Mater..

[3] Li K, Stockman MI, Bergman DJ (2003). Self-similar chain of metal nanospheres as an efficient nanolens. Phys. Rev. Lett..

[4] Stockman MI (2004). Nanofocusing of optical energy in tapered plasmonic waveguides. Phys. Rev. Lett..

[5] Merlin R (2007). Radiationless electromagnetic interference: evanescent-field lenses and perfect focusing. Science.

[6] Wang F (2016). Scanning superlens microscopy for non-invasive large field-of-view visible light nanoscale imaging. Nat. Commun..

[7] Wang F (2016). Three-dimensional super-resolution morphology by near-field assisted white-light interferometry. Sci Rep..

[8] Dürig U, Pohl DW, Rohner F (1986). Near-field optical-scanning microscopy. J. Appl. Phys..

[9] Hell SW, Wichmann J (1994). Breaking the diffraction resolution limit by stimulated emission: stimulated-emission-depletion fluorescence microscopy. Opt. Lett..

[10] Dyba M, Jakobs S, Hell SW (2003). Immunofluorescence stimulated emission depletion microscopy. Nat. Biotechnol..

[11] Betzig E (2006). Imaging intracellular fluorescent proteins at nanometer resolution. Science.

[12] Shroff H, Galbraith CG, Galbraith JA, Betzig E (2008). Live-cell photoactivated localization microscopy of nanoscale adhesion dynamics. Nat. Methods..

[13] Rust MJ, Bates M, Zhuang X (2006). Sub-diffraction-limit imaging by stochastic optical reconstruction microscopy (storm). Nat. Methods..

[14] Huang B, Jones, Sara A, Brandenburg B, Zhuang X (2008). Whole-cell 3d storm reveals interactions between cellular structures with nanometer-scale resolution. Nat. Methods..

[15] Schermelleh L (2008). Subdiffraction multicolor imaging of the nuclear periphery with 3d structured illumination microscopy. Science.

[16] Abdeladim L (2019). Multicolor multiscale brain imaging with chromatic multiphoton serial microscopy. Nat. Commun..

[17] Zhang Y (2020). Nanoscale subcellular architecture revealed by multicolor three-dimensional salvaged fluorescence imaging. Nat. Methods..

[18] Diao J, Yuan W, Yu Y, Zhu Y, Wu Y (2016). Controllable design of super-oscillatory planar lenses for sub-diffraction-limit optical needles. Opt. Express..

[19] Yuan G, Rogers ETF, Zheludev NI (2017). Achromatic super-oscillatory lenses with sub-wavelength focusing. Light-Sci. Appl..

[20] Rogers TF (2012). A super-oscillatory lens optical microscope for subwavelength imaging. Nat. Mater..

[21] Yuan G (2016). Quantum super-oscillation of a single photon. Light-Sci. Appl..

[22] Li M, Li W, Li H, Zhu Y, Yu Y (2017). Controllable design of super-oscillatory lenses with multiple sub-diffraction-limit foci. Sci Rep..

[23] Huang K (2014). Optimization-free superoscillatory lens using phase and amplitude masks. Laser Photon. Rev..

[24] Rogers KS, Bourdakos KN, Yuan G, Mahajan S, Rogers ETF (2018). Optimising superoscillatory spots for far-field super-resolution imaging. Opt. Express..

[25] Tang D (2015). Ultrabroadband superoscillatory lens composed by plasmonic metasurfaces for subdiffraction light focusing. Laser Photon. Rev..

[26] Wu Z (2020). Broadband dielectric metalens for polarization manipulating and superoscillation focusing of visible light. ACS Photonics.

[27] Yu Y, Li W, Li H, Li M, Yuan W (2018). An investigation of influencing factors on practical sub-diffraction-limit focusing of planar super-oscillation lenses. Nanomaterials.

[28] Qin F (2017). A supercritical lens optical label-free microscopy: Sub-diffraction resolution and ultra-long working distance. Adv. Mater..

[29] Rogers ETF (2013). Super-oscillatory optical needle. Appl. Phys. Lett..

[30] Yuan G (2014). Planar super-oscillatory lens for sub-diffraction optical needles at violet wavelengths. Sci Rep..

[31] Chen G (2016). Generation of a sub-diffraction hollow ring by shaping an azimuthally polarized wave. Sci Rep..

[32] Chen G (2017). Planar binary-phase lens for super-oscillatory optical hollow needles. Sci Rep..

[33] Chen G (2016). Super-oscillatory focusing of circularly polarized light by ultra-long focal length planar lens based on binary amplitude-phase modulation. Sci Rep..

[34] Ye H, Qiu C, Huang K, Teng J, Yeo SP (2013). Creation of a longitudinally polarized subwavelength hotspot with an ultra-thin planar lens: Vectorial rayleigh-sommerfeld method. Laser Phys. Lett..

[35] Zhang S (2017). Synthesis of sub-diffraction quasi-non-diffracting beams by angular spectrum compression. Opt. Express..

[36] Li W, Yu Y, Yuan W (2019). Flexible focusing pattern realization of centimeter-scale planar super-oscillatory lenses in parallel fabrication. Nanoscale.

[37] Li W, He P, Yuan W, Yu Y (2020). Efficiency-enhanced and sidelobe-suppressed super-oscillatory lenses for sub-diffraction-limit fluorescence imaging with ultralong working distance. Nanoscale.

[38] Distel M, Babaryka A, Köster RW (2006). Multicolor in vivo time-lapse imaging at cellular resolution by stereomicroscopy. Dev Dyn.

[39] Favreau P (2014). Thin-film tunable filters for hyperspectral fluorescence microscopy. J Biomed Opt..

[40] Gui C, Cui DX (2012). Functionalized gold nanorods for tumor imaging and targeted therapy. Cancer Biol Med..

[41] Poon TC, Motamedi M (1987). Optical/digital incoherent image processing for extended depth of field. Appl. Optics.

[42] Poon, T. C. *Digital Holography and Three-Dimensional Display* (Springer US, 2006).

[43] Presutti F, Monticone F (2020). Focusing on bandwidth: achromatic metalens limits. Optica.

[44] Chen G, Wen Z, Qiu C (2019). Superoscillation: From physics to optical applications. Light-Sci. Appl..

[45] Huang K, Fei Q, Hong L, Ye H, Teng J (2018). Planar diffractive lenses: fundamentals, functionalities, and applications. Adv. Mater..

[46] Born, M., Wolf, E., & Bhatia, A. B. in *Principles of Optics: Electromagnetic Theory of Propagation, Interference and Diffraction of Light* 7th edn (eds Wolf E., Born M.) (Cambridge University Press, 1999).

[47] Mahou P, Vermot J, Beaurepaire E, Supatto W (2014). Multicolor two-photon light-sheet microscopy. Nat. Methods..

[48] Li Z (2018). Achromatic broadband super-resolution imaging by super-oscillatory metasurface. Laser Photon. Rev..

[49] Konak A, Coit DW, Smith AE (2006). Multi-objective optimization using genetic algorithms: a tutorial. Reliab. Eng. Syst. Saf..

[50] Fonseca CM, Fleming PJ (1998). Multiobjective optimization and multiple constraint handling with evolutionary algorithms. I. A unified formulation. IEEE Trans. Syst. Man Cybern. A Syst. Hum..

[51] Fan Y (2020). Phase-controlled metasurface design via optimized genetic algorithm. Nanophotonics.

[52] Siegman AE (1977). Quasi fast hankel transform. Opt. Lett..

[53] Brown M, Lowe DG (2007). Automatic panoramic image stitching using invariant features. Int. J. Comput. Vis..

[54] Rublee, E., Rabaud, V., Konolige, K., Bradski, G. Orb: An efficient alternative to sift or surf. In: *2011 International Conference on Computer Vision* (IEEE, 2011).

[55] Rosten, E., Drummond, T. in *Computer Vision—ECCV 2006* (eds Leonardis, A., Bischof, H. & Pinz, A.) (Springer Berlin Heidelberg, 2006).

[56] Bay H, Ess A, Tuytelaars T, Van Gool L (2008). Speeded-up robust features (surf). Comput. Vis. Image Underst..

[57] Lowe DG (2004). Distinctive image features from scale-invariant keypoints. Int. J. Comput. Vis..

[58] Beis, J. S., Lowe, D. G. Shape indexing using approximate nearest-neighbour search in high-dimensional spaces. In *Proc. IEEE Comput. Soc. Conf. Comput. Vis. Pattern Recognit.*10.1109/CVPR.1997.609451 (1997).

[59] Fischler, M. A. & Bolles, R. C. in: *Readings in Computer Vision* (eds Fischler M. A., Firschein O.) (Morgan Kaufmann, 1987).

[60] Li J, Wang Z, Lai S, Zhai Y, Zhang M (2018). Parallax-tolerant image stitching based on robust elastic warping. IEEE Transactions on Multimedia.

