# Author Correction: Super-resolution multicolor fluorescence microscopy enabled by an apochromatic super-oscillatory lens with extended depth-of-focus

**DOI:** 10.1038/s41467-024-45994-6

**Published:** 2024-02-19

**Authors:** Wenli Li, Pei He, Dangyuan Lei, Yulong Fan, Yangtao Du, Bo Gao, Zhiqin Chu, Longqiu Li, Kaipeng Liu, Chengxu An, Weizheng Yuan, Yiting Yu

**Affiliations:** 1 Ningbo Institute of Northwestern Polytechnical University, College of Mechanical Engineering, Northwestern Polytechnical University, Xi’an, 710072 China; 2 Key Laboratory of Micro/Nano Systems for Aerospace (Ministry of Education), Northwestern Polytechnical University, Xi’an, 710072 China; 3 Shaanxi Province Key Laboratory of Micro and Nano Electro-Mechanical Systems, Northwestern Polytechnical University, Xi’an, 710072 China; 4 Department of Materials Science and Engineering, City University of Hong Kong, Hong Kong, 999077 China; 5 The Institute of AI and Robotics, Fudan University, Shanghai, 200433 China; 6 Key Laboratory of Spectral Imaging Technology of Chinese Academy of Sciences, Xi’an Institute of Optics and Precision Mechanics, Chinese Academy of Sciences, Xi’an, 710119 China; 7 Department of Electrical and Electronic Engineering, Joint Appointment with School of Biomedical Sciences, The University of Hong Kong, Hong Kong, 999077 China; 8 State Key Laboratory of Robotics and System, Harbin Institute of Technology, Harbin, Heilongjiang 150001 China

**Keywords:** Imaging and sensing, Biophotonics

Correction to: *Nature Communications* 10.1038/s41467-023-40725-9, published online 22 August 2023

The original version of this Article contained an error in Page 2, Introduction, which incorrectly read ‘which reveals a far-field resolution limit of 0.3λ at 488 nm.’ The correct version states ‘0.53λ at 488 nm’ in place of ‘0.3 λ at 488 nm’. This has been corrected in both the PDF and HTML versions of the Article.

The original version of this Article contained an error in Page 4, Results, which incorrectly read ‘The center to center (CTC) distances of the resolution testing chart are set as 150 nm, 250 nm and 350 nm, respectively, as shown in Fig. 3b.’ The correct version states ‘resolution testing chart are 258 nm, 456 nm and 677 nm, respectively, as shown in Fig. 3b.’ in place of ‘resolution testing chart is set as 150 nm, 250 nm and 350 nm, respectively, as shown in Fig. 3b.’. This has been corrected in both the PDF and HTML versions of the Article.

The original version of this Article contained an error in Page 4, Results, which incorrectly read ‘…and even the 150 nm CTC distance can be resolved under 488 nm illumination (Fig. 3f).’ The correct version states ‘…and even the 258 nm CTC distances’ in place of ‘…and even the 150 nm CTC distance’. This has been corrected in both the PDF and HTML versions of the Article.

The original version of this Article contained an error in Page 4, Results, which incorrectly read ‘…our SOL-based microscope exhibits a CTC resolution of 150 nm (i.e., 0.30λ at λ=488 nm).”’ The correct version states ‘…our SOL-based microscope exhibits a resolution of 258 nm (i.e., 0.53λ at λ = 488 nm).’ in place of ‘…our SOL-based microscope exhibits a CTC resolution of 150 nm (i.e., 0.30 λ at λ = 488 nm)’. This has been corrected in both the PDF and HTML versions of the Article.

The Page 2, Introduction originally incorrectly read ‘…with a focusing efficiency of 11.2%, 12% and 21.5% respectively at a focal distance of 428 μm, far surpassing the results in Ref. ^19^ and other reports^37^’ The correct version states ‘with a side-lobe suppression ratio of 10.13 dB, 10.66 dB and 9.32 dB, respectively at a focal distance of 428 μm, surpassing all results published in the literature^20, 21, 29, 30, 33, 37^’ instead of ‘with a focusing efficiency of 11.2%, 12% and 21.5% respectively at a focal distance of 428 μm, far surpassing the results in Ref. ^19^ and other reports^37^’. This has been corrected in both the PDF and HTML versions of the Article.

The Page 4, Results originally incorrectly read ‘such as the focusing efficiency (over 11.2% at three wavelengths), is additionally presented, which surpasses the best reported apochromatic SOL^19^’ The correct version states ‘such as the side-lobe suppression ratio over 9.3 dB, is additionally presented, which surpasses the best reported apochromatic SOL with an extended depth-of-focus’ instead of ‘such as the focusing efficiency (over 11.2% at three wavelengths), is additionally presented, which surpasses the best reported apochromatic SOL^19^’. This has been corrected in both the PDF and HTML versions of the Article.

The Page7, Discussion originally incorrectly read ‘At the incident wavelength of 488 nm, the 150 nm-width nanoslits can be easily distinguished, in contrast to the blurred images’ The correct version states ‘the 258 nm-width nanoslits’ instead of ‘the 150 nm-width nanoslits’, is additionally presented, which surpasses the best reported apochromatic SOL^19^’. This has been corrected in both the PDF and HTML versions of the Article.

The original version of this Article contained an error in Page 10, Preparation of cell samples, which incorrectly read ‘The Zhejiang HopCell biological company ...’ The correct version states ‘Zhejiang Hopstem Bioengineering company limited’ in place of ‘Zhejiang HopCell biological company’. This has been corrected in both the PDF and HTML versions of the Article.

The original version of the Supplementary Information associated with this Article contained errors in lines 117–118, page 6, ‘The Zhejiang HopCell biological company provided the services of cell differentiation …’ The correct version states ‘Zhejiang Hopstem Bioengineering Co. Ltd. provided the services of the DYR0100 cell differentiation ...’ in place of ‘The Zhejiang HopCell biological company provided the services of cell differentiation …’. The HTML has been updated to include a corrected version of the Supplementary Information.

The original version of Supplementary Information associated with this Article contained errors in Supplementary Table 1 (lines 203–205, page 18), where Ref. ^20^ should be replaced by Ref. ^30^, Ref. ^23^ by Ref. ^18^, and Ref. ^18^ by Ref. ^19^. The HTML has been updated to include a corrected version of Supplementary Information.

The original version of the Supplementary Information associated with this Article contained errors in Supplementary Table 2. The HTML has been updated to include a corrected version of the Supplementary Information; the original incorrect versions of these Figures can be found as Supplementary Information associated with this Correction.

The original version of this Article contained an error in Fig. 3b, in which the resolvable CTC distances are incorrect. This has been corrected in both the PDF and HTML versions of the Article.

The original version of this Article contained an error in Fig. 3f–h, in which the resolvable CTC distances are incorrect. This has been corrected in both the PDF and HTML versions of the Article.

### Supplementary information

Original Supplementary Information

Updated Supplementary Information

