# Supplementary material for: Super-resolution multicolor fluorescence microscopy enabled by an apochromatic super-oscillatory lens with extended depth-of-focus
Source: Nat Commun. 2023 Aug 22;14:5107. doi: 10.1038/s41467-023-40725-9 (PMC10444772; doi:10.1038/s41467-023-40725-9)
Supplement: Supplementary file 2 — Description of Additional Supplementary Files [file 41467_2023_40725_MOESM2_ESM.docx]

**Description of Additional Supplementary Files**

**Supplementary Movie 1:**

The cucoloris of the scanning imaging process for the resolution test target at the illumination of λ=640 nm.

**Supplementary Movie 2:**

The cucoloris of the scanning imaging process for the neuron cell at the illumination of λ=488 nm.
