# Supplementary material for: Author Correction: Super-resolution multicolor fluorescence microscopy enabled by an apochromatic super-oscillatory lens with extended depth-of-focus
Source: Nat Commun. 2024 Feb 19;15:1510. doi: 10.1038/s41467-024-45994-6 (PMC10876544; doi:10.1038/s41467-024-45994-6)
Supplement: Supplementary file 2 — Updated Supplementary Information [file 41467_2024_45994_MOESM2_ESM.pdf]

Supplementary Information

Super-resolution multicolor fluorescence microscopy enabled by an apochromatic super-oscillatory lens with extended depth-of-focus

Wenli Li,[1,2,3] Pei He,[1,2,3] Dangyuan Lei[4]* Yulong Fan,[4] Yangtao Du,[5] Bo Gao,[6] Zhiqin Chu,[7] Longqiu Li,[8] Kaipeng Liu,[8] Chengxu An,[1,2,3] Weizheng Yuan,[1,2,3] and Yiting Yu,[1,2,3]*

[1] Ningbo Institute of Northwestern Polytechnical University, College of Mechanical Engineering, Northwestern Polytechnical University, Xi'an 710072, China

[2]Key Laboratory of Micro/Nano Systems for Aerospace (Ministry of Education), Northwestern Polytechnical University, Xi'an 710072, China

[3]Shaanxi Province Key Laboratory of Micro and Nano Electro-Mechanical Systems, Northwestern Polytechnical University, Xi'an 710072, China

[4]Department of Materials Science and Engineering, City University of Hong Kong, Hong Kong 999077, China

[5]The Institute of AI and Robotics, Fudan University, Shanghai 200433, China

[6]Key Laboratory of Spectral Imaging Technology of Chinese Academy of Sciences, Xi'an Institute of Optics and Precision Mechanics, Chinese Academy of Sciences, Xian 710119, China

[7]Department of Electrical and Electronic Engineering, Joint Appointment with School of Biomedical Sciences, The University of Hong Kong, Hong Kong 999077, China

[8]State Key Laboratory of Robotics and System, Harbin Institute of Technology, Harbin, Heilongjiang 150001, China

Email: dangylei@cityu.edu.hk; yyt@nwpu.edu.cn

Supplementary Note 1. Genetic algorithm optimization

The flow chart of genetic algorithm (GA) optimization processes is shown in Supplementary Fig. 1. First, to launch the process, random phase masks are created as initial populations. At the same time, objective functions with prescribed focal lengths at different wavelengths are defined. The objective functions serve as the fitness value. Second, the position and intensity of the customized multifoci along the optical axis are initialized. The number of focal spots must be sufficient to satisfy the requirements for generating a needle-like focusing region. To construct an unconstrained optimization process, the intensity modulation of the needle-like optical pattern formed by bridging the axial multifoci is added as penalty function terms, so that the objective functions are divergence-free. Third, genetic operations among the populations are iteratively carried out, including screened selection, crossover and mutation, and the offspring populations to the original parental populations are generated according to the fitness value of each mask until the termination criterion is satisfied. Finally, an optimized phase distribution profile for an apochromatic super-oscillatory lens (SOL) is found as shown in Supplementary Fig. 2, and optimized results are given in Supplementary Table 2. We can also create an SOL possessing a longer DoF of 23.4λ optimized at 640 nm through our algorithm is unveilled in Supplementary Fig. 3, with its optical characteristics is shown in Supplementary Table 3. To test the possibility of our proposed algorithm to create apochromatic SOL with higher NA, we increase the diameter of the SOL to 1.2 mm and the working distance (WD) at 395 nm, equivalent to a numerical aperture (NA) of 0.84. The reason that we cannot push it to a larger one is to balance the focusing property of SOL and the

computing resource. To save the simulation time, only two incident wavelengths of 488 nm and 532 nm are optimized. The calculated results are revealed in Supplementary Fig. 4.

Supplementary Note 2. Wafer-scale fabrication of the achromatic SOL

First, a Si_xN_y layer is deposited on a 4-inch Pyrex glass substrate by plasma-enhanced chemical vapor deposition (PECVD), followed by a standard optical lithography. Then, reactive ion etching (RIE) is employed to form the prescribed non-subwavelength Si_xN_y ring belts. The optical properties of the Si_xN_y wafer deposited via plasma-enhanced chemical vapor deposition (PECVD) are measured by spectroscopic ellipsometry (ELLITOP Scientific Co., Ltd. ES01). The measured refractive index at 532 nm is n_{532}=2.25 as shown in Supplementary Fig. 4. The ring belts are perforated on a 213 nm-thick Si_xN_y film to achieve a phase difference of π.

Supplementary Note 3. Experimental validation of far-field focusing

The single wavelength of the far-field focusing characterization measurement is provided by the fiber-coupled lasers at 488 nm, 532 nm, and 640 nm, respectively. A customized fiber-optic coupled multiplexer is used to combine the three incident wavelengths to ensure precise alignment of the incident angle. As illustrated in Supplementary Fig. 6, after being collimated by a fiber coupler, the beam is directed into a customized inverted microscope (Nikon Eclipse Ti-U) by using several optical components and then illuminates the SOL. We use a high-magnification, high-numerical aperture objective (Nikon, TU Plan Fluor 100X, NA = 0.9, Working Distance 1 mm) to collect the diffracted beams which are subsequently imaged by a high-resolution camera (Olympus,

2560×1280, the mesh size in the image processing software is 0.3 μm) slice by slice with an axial step of 20 nm. The transverse cross-sectional distributions in the propagation direction are obtained through z scanning on the SOL mounted on the piezo stage (Physik Instrument, E-816). The longitudinal cross-sectional distributions are formed from built-in data processing software ImageJ 1.47v.

Supplementary Note 4. Investigating the uniformity of focusing pattern

The curves of the measured full width at half maximums (FWHMs) along the optical axis for three incident wavelengths are plotted to further investigate the uniformity of the designed optical needle-like focusing pattern. The corresponding results are illustrated in Supplementary Fig. 7. The FWHM for five typical points within the focusing region at discrete axial distances are extracted and marked as blue asterisks in bold for all the three incident wavelengths for further comparison.

Supplementary Note 5. Sub-diffraction multicolor 2D imaging

To verify the resolving capability of the customized apochromatic SOL-based microscopy, the resolution target prepared by the focused ion beam (FIB) milling is used as a label-free imaging sample. Light scattered by the object is collected by a high-NA (NA = 0.9) objective lens with a 1 mm-long working distance in air. Finally, the measured images of the target are projected onto a CCD camera. The whole imaging equipment is built up on the basis of the inverted fluorescent microscopy with long working distance fluorescent objective (Nikon, TU Plan Fluor 100X, NA = 0.9, working distance 1 mm), which is sketched in Supplementary Fig. 8. The imaging samples are situated at the focal plane of the SOLs and

raster scanned in *x-y* plane relative to the focal spot during the imaging process by a piezo-stage (SYMC, DZNS-X200Y200Z100-01). The samples are scanned at a step size of 20 nm along the X and Y axis, respectively. The collected signal is recorded by a high-speed real-time splicing camera (Tucsen MIchrome 5 Pro) at 35 fps with the built-in advanced imaging calculated processing software Mosaic 2.1. For the confocal imaging part, the resolution chart was tested in the laser scanning confocal microscopy (LSCM, Leica DMi8) under the illumination wavelength of 488 nm, 552 nm and 638 nm.

Supplementary Note 6. Scanning-free 3D imaging

The 3D metallic wedge is prepared by FIB as well. After thoroughly cleaning the substrate, the multilayers of Cr thin film is deposited by magnetron sputtering on the glass substrate, resulting in a total layer stack with a total thickness of 1.3 μm. The fishnet pattern, which is composed of 1 μm period array of 500 nm-diameter transparent holes, is fabricated on the 1.3 μm-thickness Cr film by a dual beam FIB system (FEI Scios2) at an accelerating voltage of 30KV and a milling current of 20pA with the technique of gray-scale etching. Then the optical wedge is subsequently formed by the same FIB. The final sample is imaged by using a scanning electron microscope (SEM, FEI Scios2).

The experimental characterization of the imaging capability is performed by a customized system based on an inverted microscope (Nikon Eclipse Ti-U), as schematically shown in Supplementary Fig. 8 and actual experimental setup of microscopy is shown in Supplementary Fig. 9. To compare the result of the SOL-based microscopy, the imaging results of this wedge are also tested by transmission mode microscopy (T-mode) for the wide-field imaging, laser scanning confocal microscopy (LSCM, Leica DMi8).

Supplementary Note 7. Dual-color 3D cellular imaging

The DYR0100 cells (induced pluripotent stem cells-iPSC) were offered by the research group of Prof. Cao Yi from Xiangtan University. Zhejiang Hopstem Bioengineering Co. Ltd. provided the services of the DYR0100 cells' differentiation and labeling according to the method mentioned in the published article [DOI: 10.1126/scitranslmed.aad0623], which was used for bioimaging in this study. The cells are grown on a glass slide with the size of 11 mm×11 mm×0.13 mm and then covered by a 35 mm×90 mm×0.17 mm glass to adapt the size of the customized objective table embedded in the inverted microscopy. The excitation and emission spectra of the fluorescent particles are shown in Supplementary Fig. 10. Lasers of 488 nm, 532 nm are chosen as the incident light sources to stimulate the fluorescent bio-tissues. The whole bio-imaging setup is similar to the one presented in Supplementary Fig. 8 except the resolution chart is replaced by labeled neurons. Based on the fiber-optic coupled multiplexer, the incident laser can be switched sequentially to stimulate the labeled cells. The imaging samples are situated at the focal plane of the SOLs and raster scanned in *x*-*y* plane relative to the focal spot during the imaging process by a piezo-stage (SYMC, DZNS-X200Y200Z100-01). A customized continuous scanning control program based on Visual Studio is developed to synchronously control the CCD camera and the piezo-stage, both of which can be adjusted according to the predesigned track. The samples are continuously scanned at a step size of 20 nm at the X and Y axis, respectively. The collected signal is recorded by the high-speed real-time splicing camera (Tucsen MIchrome 5 Pro).

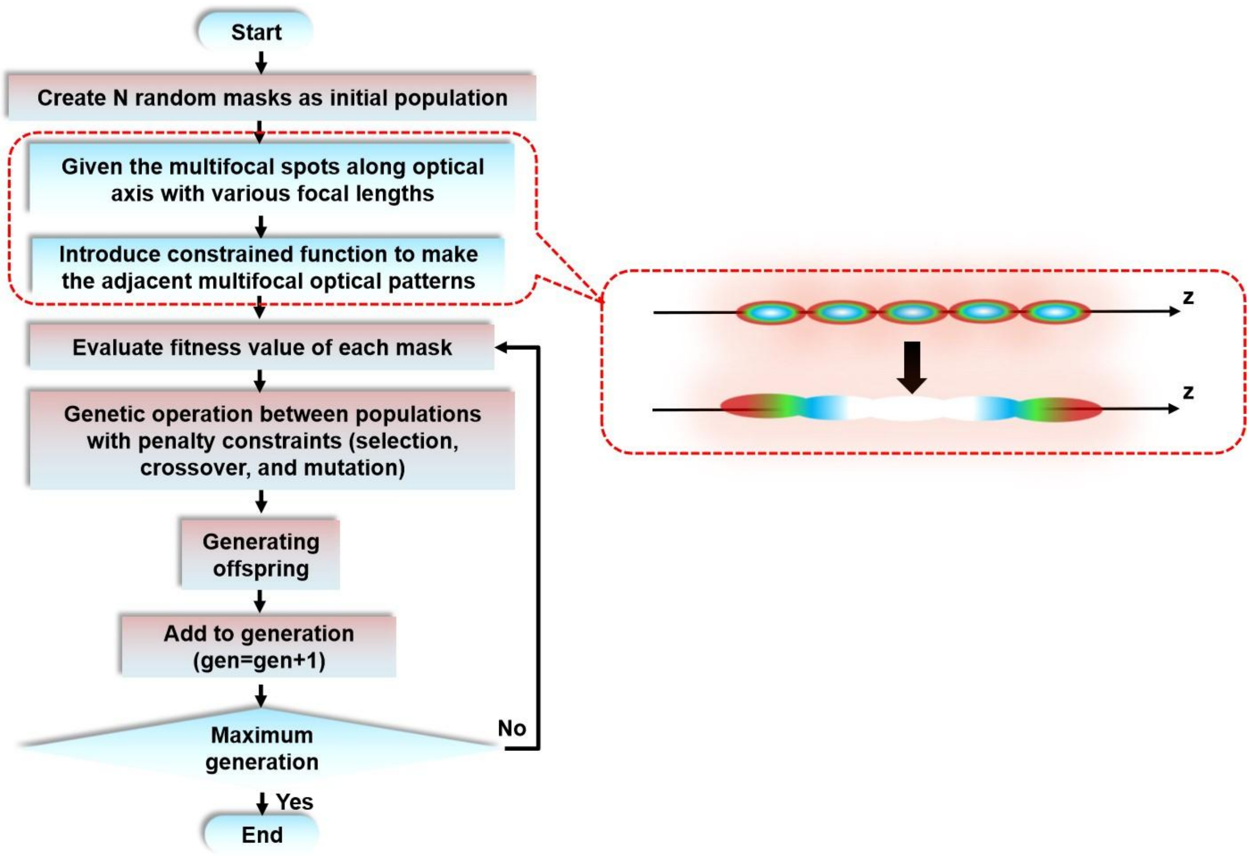

Supplementary Fig. 1 Flow Chart of the GA optimization process for generating an achromatic SOL with extended DOF, suppressed sidelobe intensity and minimized main-lobe FWHM.

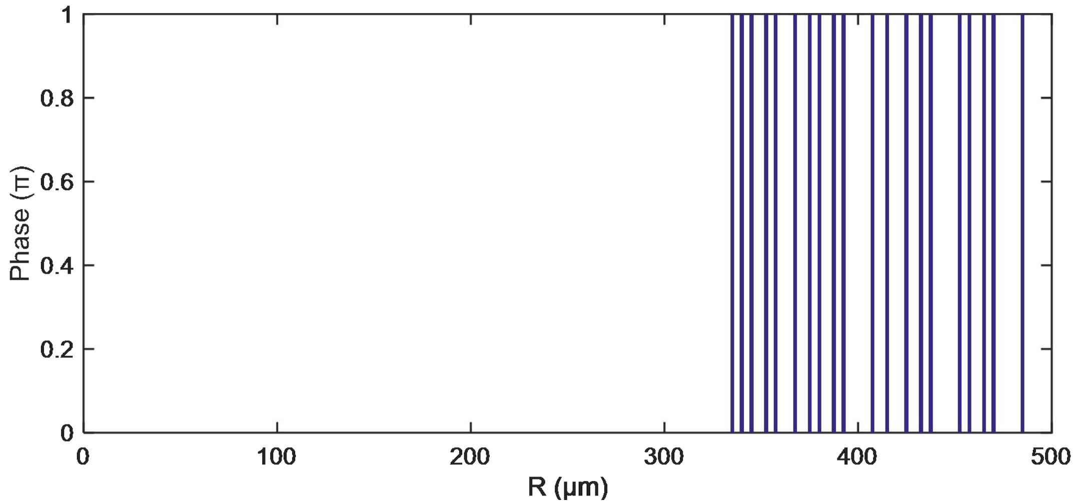

Supplementary Fig. 2 Phase distribution along the radius of the optimized phase-modulation mask.

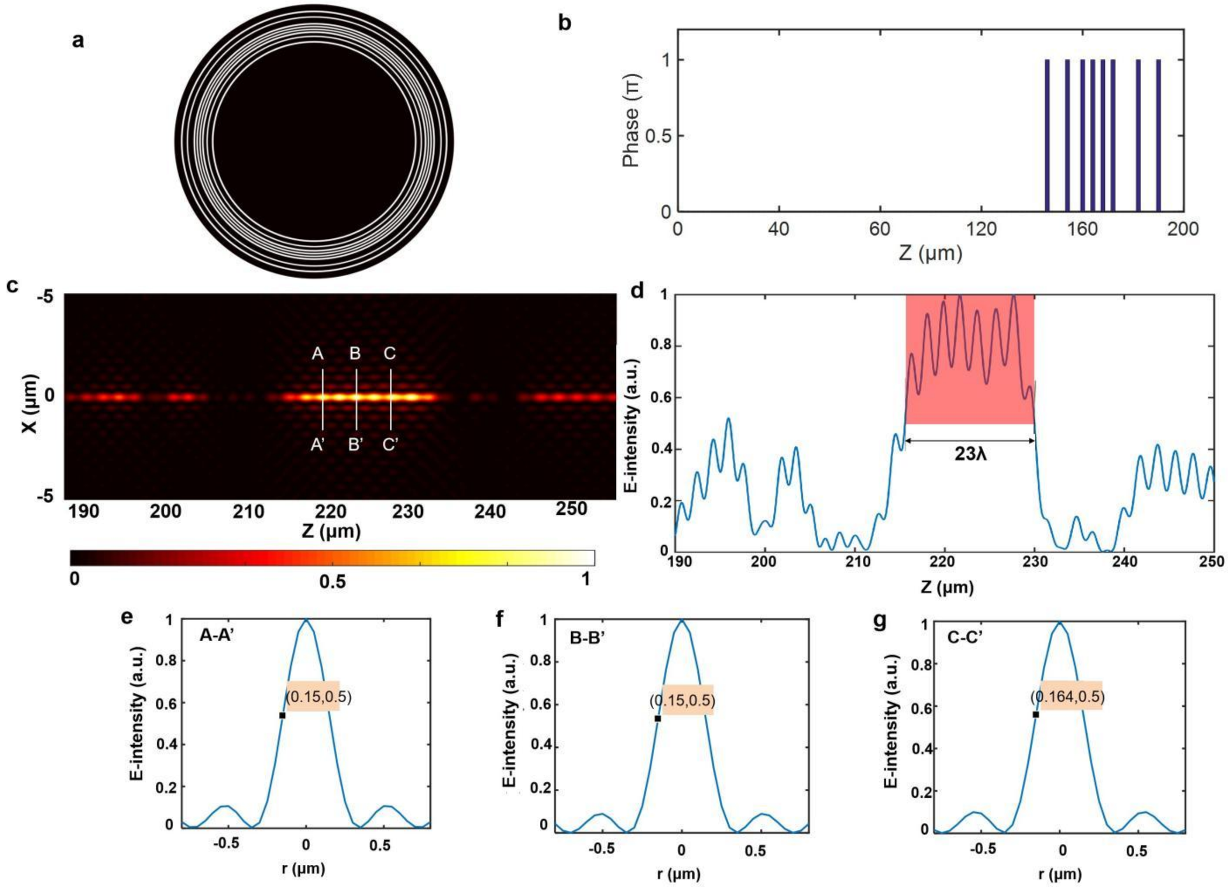

Supplementary Fig. 3. The far-field sub-diffraction focusing properties of the customized SOL with extended DoF. a Structural schematic. **b** Phase distribution. **c** Intensity pattern in XZ plane. **d** Longitudinal intensity distribution curve. **e-g** Radial intensity distribution curve at the plane AA', BB' and CC' of the customized SOL.

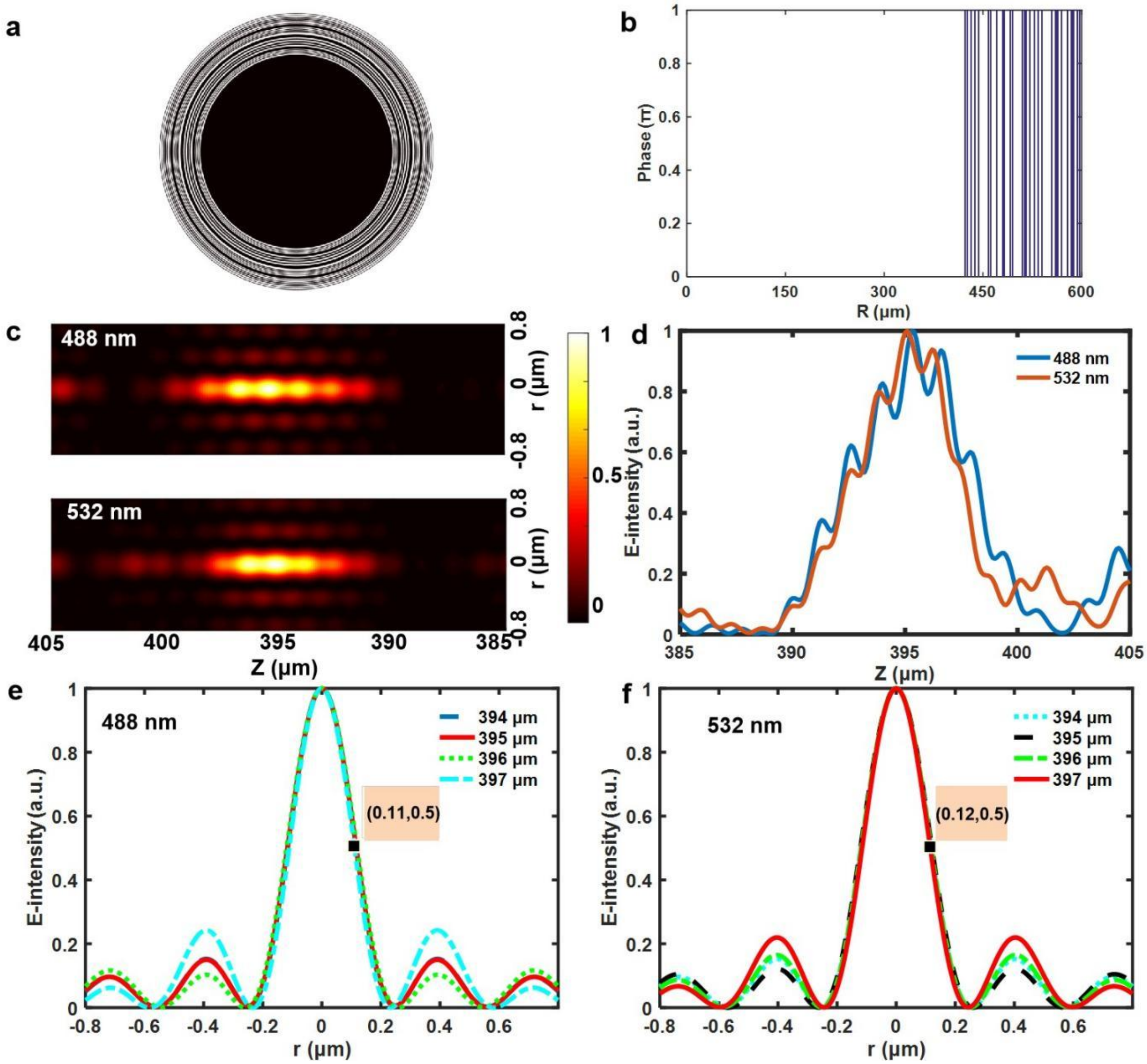

Supplementary Fig. 4 The far-field sub-diffraction focusing properties of the customized SOL with lager aperture (diameter 1.2 mm and NA=0.84). a, c Structural schematic and the corresponding phase distribution. **b** Intensity pattern in XZ plane. **d** Longitudinal intensity distribution. **e-f** Radial intensity distribution curve at the plane AA', BB' and CC' of the customized SOL. We find the FWHM and DOF at 488 nm are 220 nm and 7 μm (14.3λ), and the FWHM and DOF at 532 nm are 230 nm and 6 μm (11.3λ).

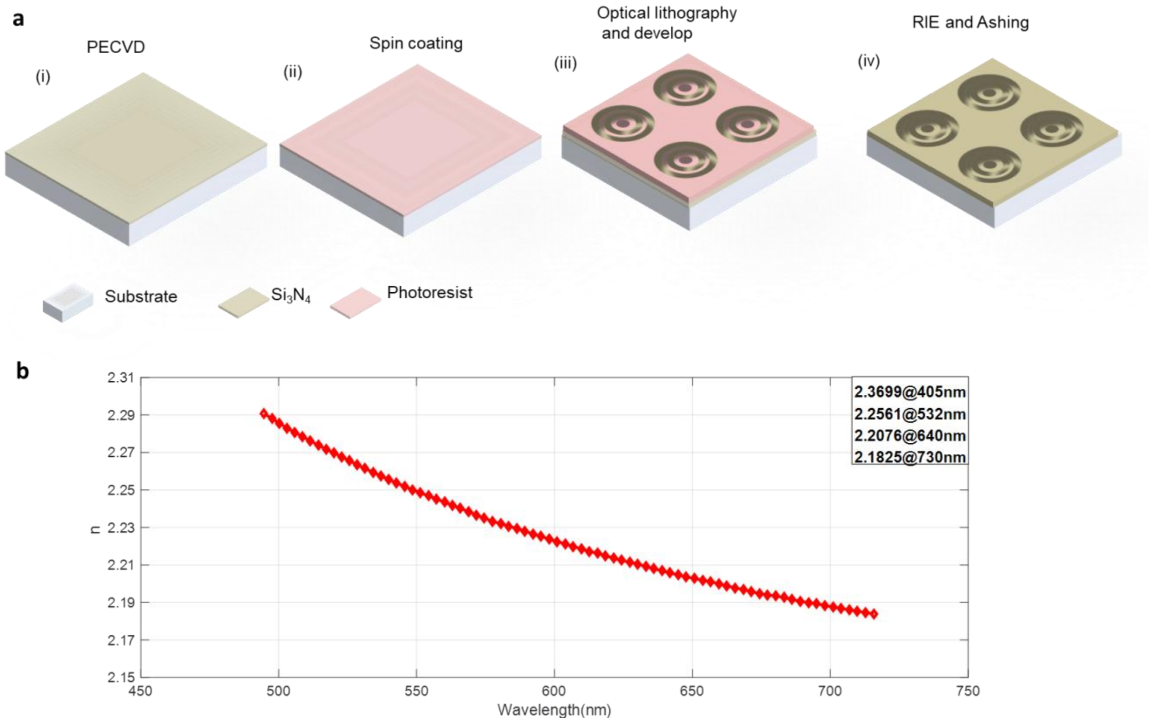

Supplementary Fig. 5 Fabrication of the achromatic SOL. a Schematics showing the wafer-level fabrication of the achromatic SOL with extended DoF. **b** Measured refractive index of fabricated SixNy wafer.

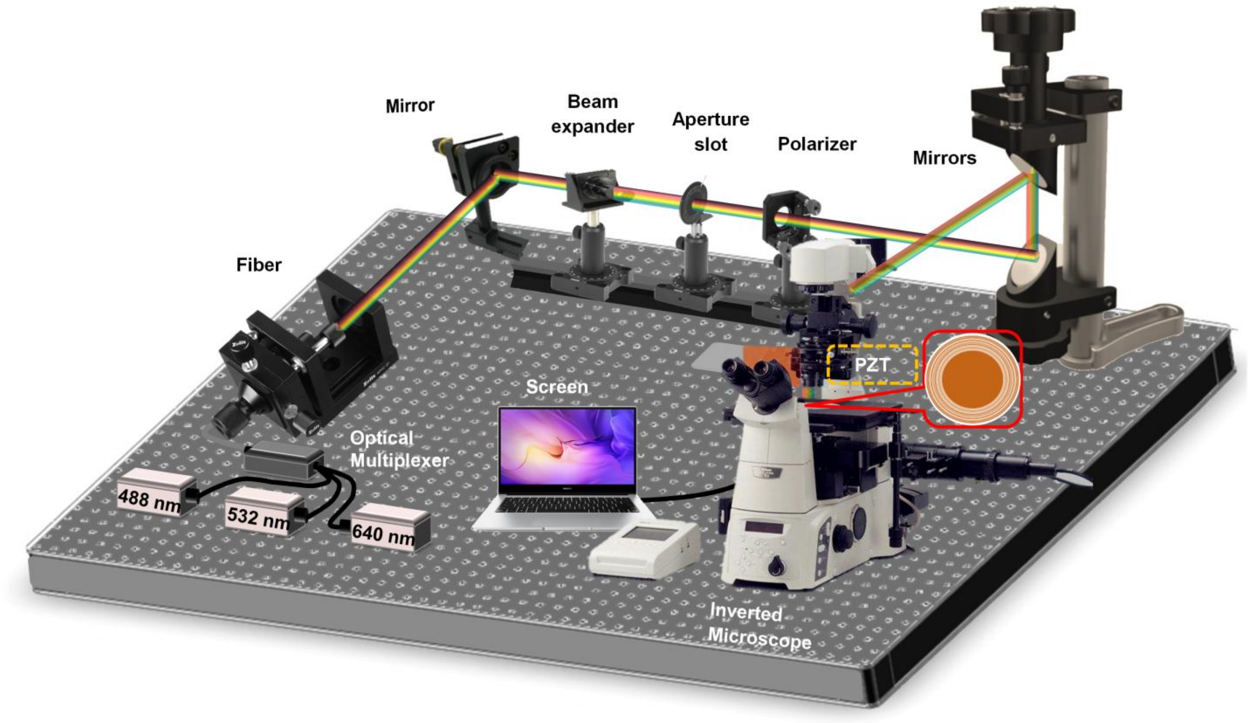

Supplementary Fig. 6 Schematic of optical measurement setup for far-field focusing properties of the apochromatic SOL.

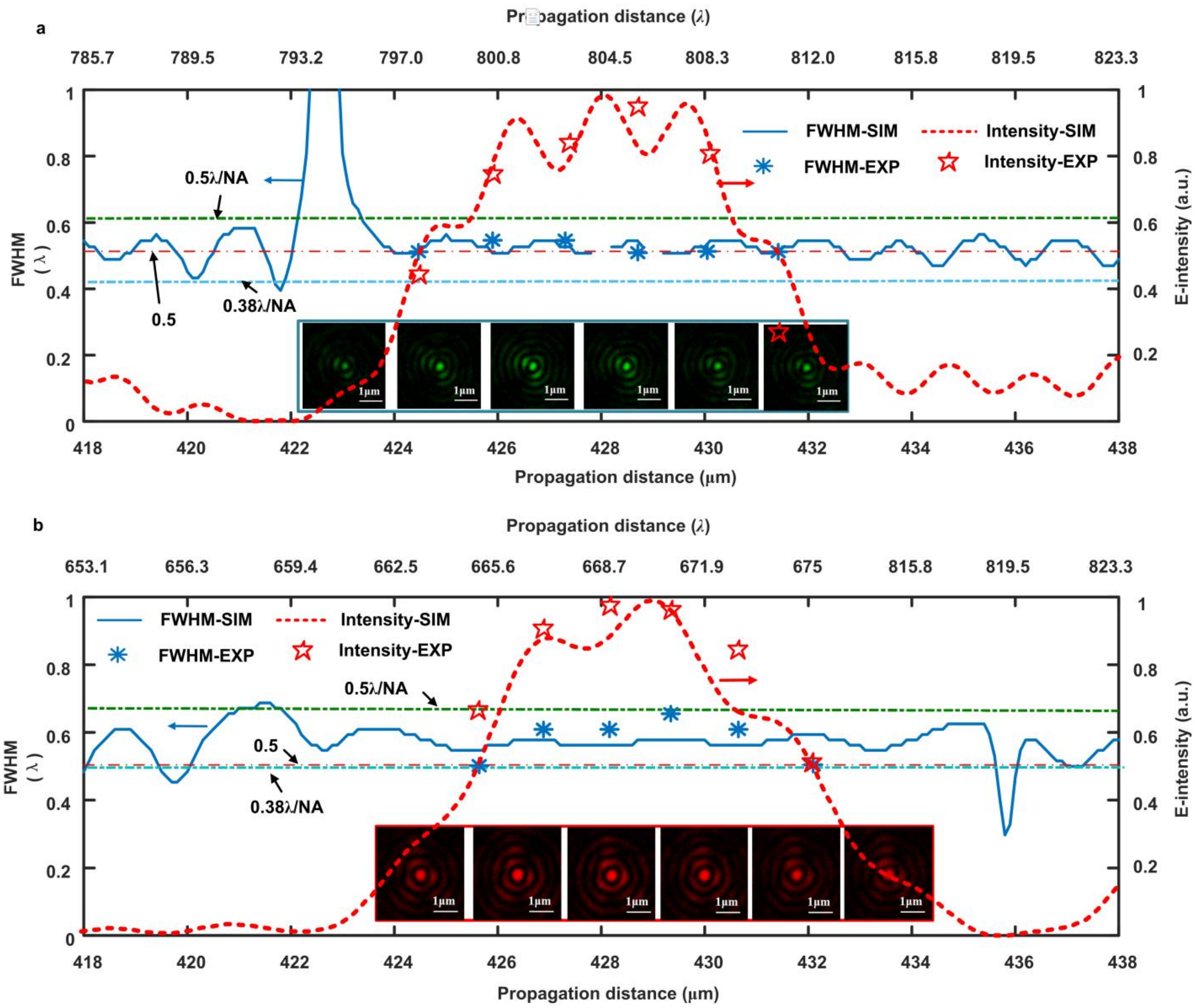

Supplementary Fig. 7 FWHM measurement at three wavelengths in the propagation direction: simulated (solid blue curve) and measured (blue asterisks) FWHMs for a λ_B, **b** λ_G, **c** λ_R, with the red dashed curve representing the simulated longitudinal optical intensity near the focal length. Insets: transverse intensity distributions from z=426 μm to z=430 μm at a step size of 1 μm for λ_B, λ_G and λ_R, respectively.

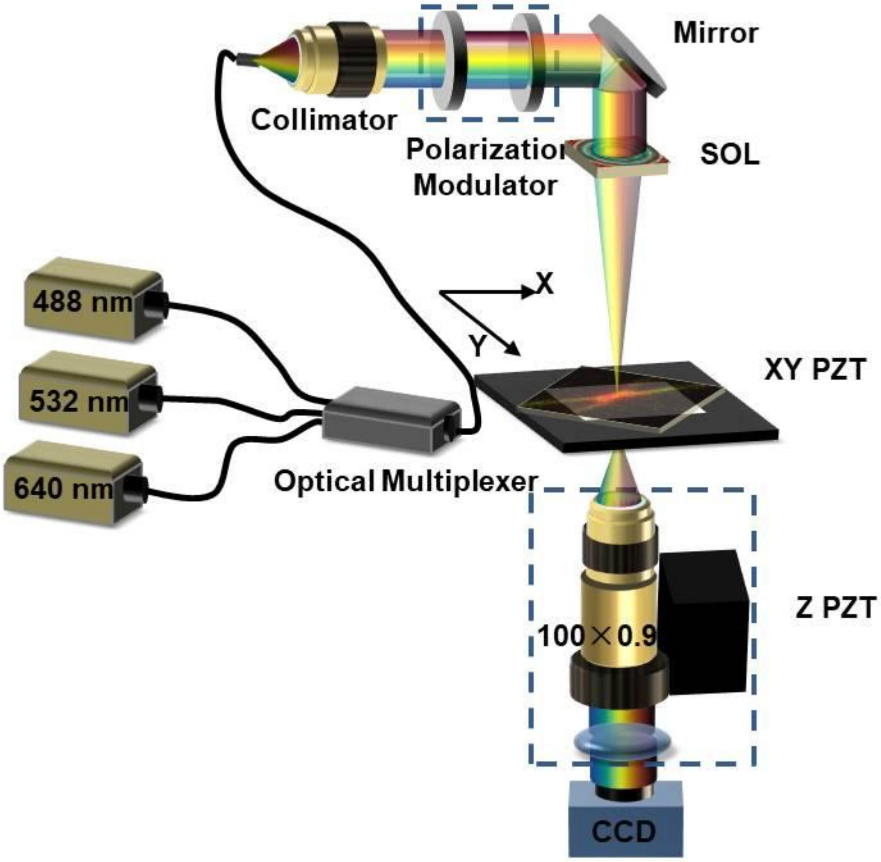

Supplementary Fig. 8 Schematic illustration of experimental setup for multi-color imaging enabled by our customized SOL integrated to an objective. PZT: Piezoelectric Transducer. Note that the resolution target should be replaced by labelled neuron cells for multi-color bio-imaging experiment.

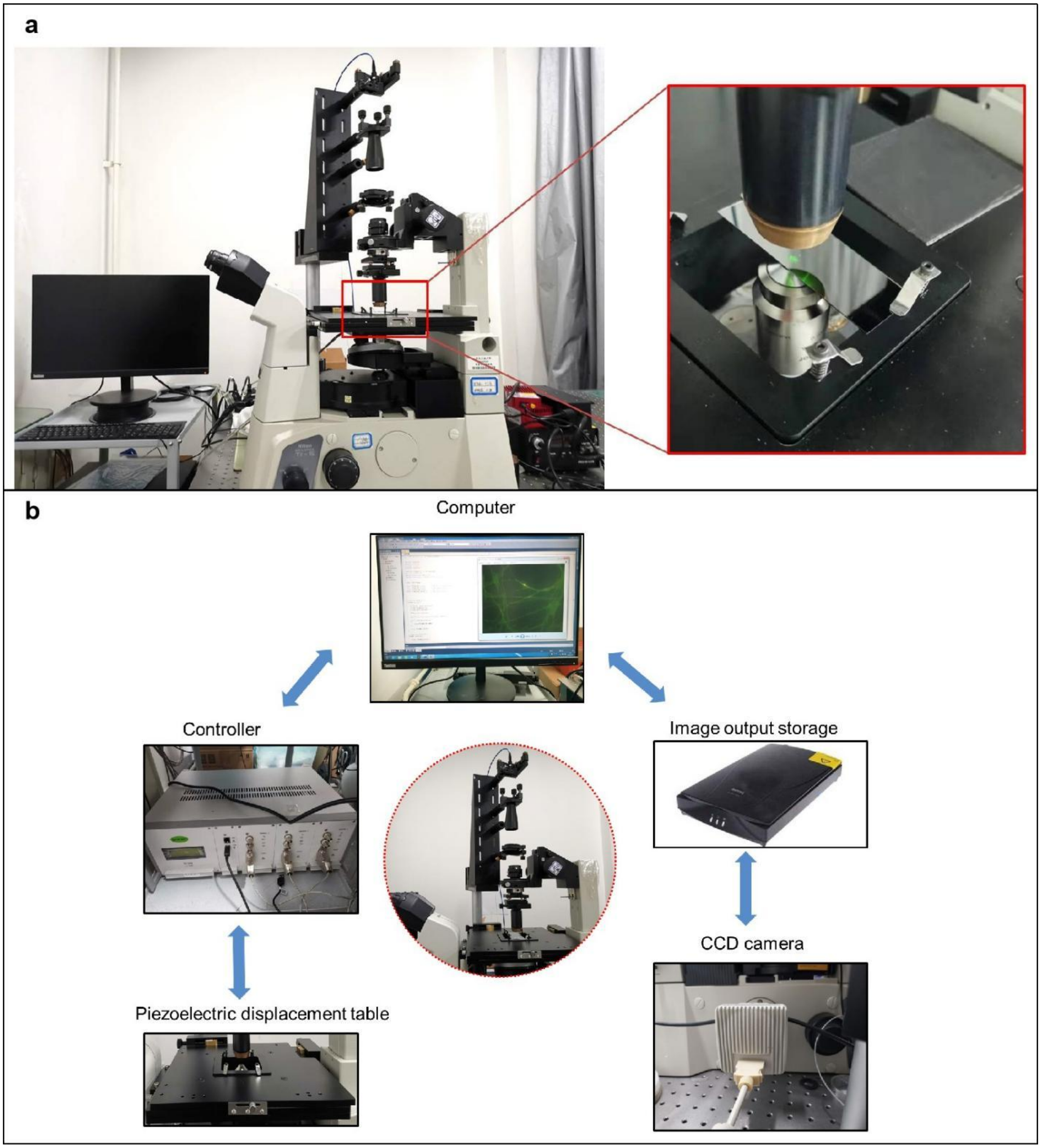

Supplementary Fig. 9 Experimental setup a The actual experimental setup of the microscopy; **b** The working scheme of current step-by-step mode.

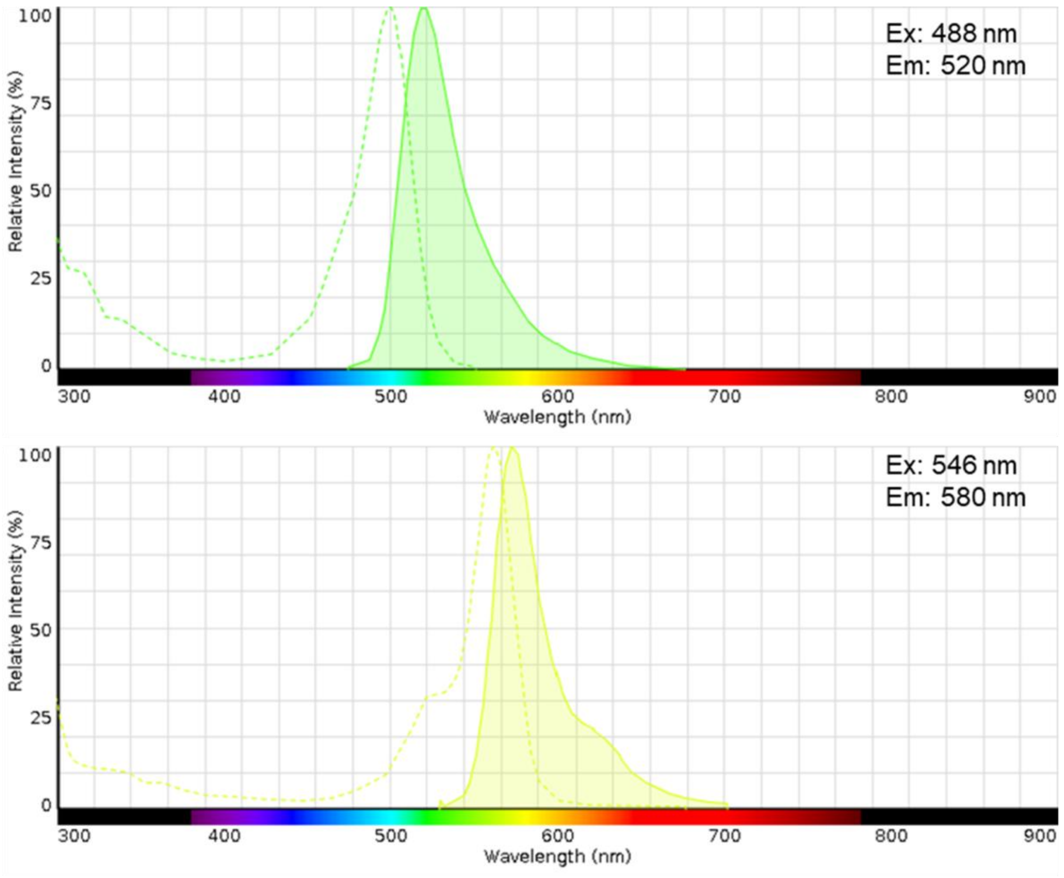

Supplementary Fig. 10 Excitation and emission spectra of the two used fluorescent dyes.

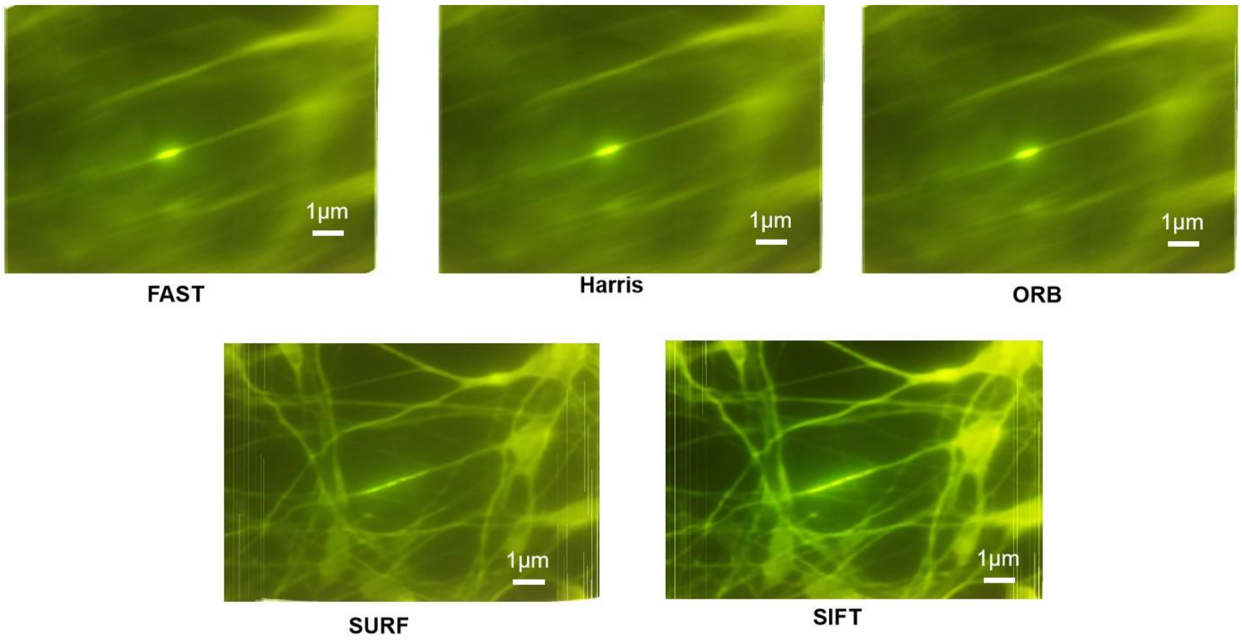

Supplementary Fig. 11 Comparison of the imaging algorithms on the reconstructed image quality.

Supplementary Table 1 Recent achievements of single hotspot focusing, monochromatic SOL with extended DoF and multi-color SOLs as compared with our work.

	Wavelength /nm	FWHM /λ	DoF /λ	Focal length /μm	Polarization	NA	Ambient medium	Refs
Monochromatic SOL with single hotspot	640	0.289	-	10	LP	1.35	Oil	Rogers et al[24]
Monochromatic SOL with extended DoF	405	0.45	15	10	CP	0.89	Air	Yuan et al[30]
	532	0.34	5	10	LP	0.89	Oil	Diao et al[18]
Achromatic focusing SOL	405<br>532<br>633	0.457<br>0.445<br>0.54	-	10	LP	0.89	Air	Yuan et al[19]
**Achromatic SOL with extended DOF**	**488**<br>**532**<br>**640**	**0.50**<br>**0.50**<br>**0.56**	**15**<br>**12.7**<br>**10.2**	**428**	**LP**	**0.76**	**Air**	**This work**

Notes: LP, linear polarization; CP, circular polarization.

Supplementary Table 2 Key parameters of the designed apochromatic SOL with extended DoF

R (μm)	λ (nm)	Focal length (μm)	DoF (μm)				side-lobe suppression ratio	Spot size (nm)	
			Theory	Sim	Exp	$V = \frac{DoF_{Sim}}{DoF_{Theory}}$		Sim	Exp
	λ_B=488		1.4	7.2	7.3	5.1	10.13 dB	245	256
500	λ_G=532	428	1.5	6.9	6.8	4.6	10.66 dB	270	270
	λ_R=640		1.8	6.6	6.5	3.7	9.32 dB	360	390

Supplementary Table 3 Key parameters of a customized SOL with a 23.4λ long DoF

R(μm)	λ (nm)	Focal length (μm)	NA	DoF (μm)			Spot size (nm)	
				DoF_{Theory}	DoF_{Sim}	$V = \frac{DoF_{Sim}}{DoF_{Theory}}$	Sim	Abbe limit
300	λ=640	220	0.81	1.55	15	8	320	395

Supplementary Table 4 Key parameters of the designed apochromatic SOL working at air and cover glass.

R (μm)	λ (nm)	DoF (μm)			Spot size (nm)		
		Theory (Air)	Sim (Air)	Air + Cover glass + water	Theory (Air)	Sim (Air)	Air + Cover glass + water
500	λ_B=488	1.4	7.2	9	321	245	170
	λ_G=532	1.5	6.9	8.1	350	270	200
	λ_R=640	1.8	6.6	6.8	421	360	320
